# Lung ventilation strategies for acute respiratory distress syndrome: a systematic review and network meta-analysis

**DOI:** 10.1038/srep22855

**Published:** 2016-03-09

**Authors:** Changsong Wang, Xiaoyang Wang, Chunjie Chi, Libo Guo, Lei Guo, Nana Zhao, Weiwei Wang, Xin Pi, Bo Sun, Ailing Lian, Jinghui Shi, Enyou Li

**Affiliations:** 1 Department of Anesthesiology, the First Affiliated Hospital of Harbin Medical University, Harbin, China; 2 Department of critical care medicine, the Third Affiliated Hospital of Harbin Medical University, Harbin, China; 3 Department of Anesthesiology, JILIN GUO WEN Hospital, Gongzhuling, China

## Abstract

To identify the best lung ventilation strategy for acute respiratory distress syndrome (ARDS), we performed a network meta-analysis. The Cochrane Central Register of Controlled Trials, EMBASE, MEDLINE, CINAHL, and the Web of Science were searched, and 36 eligible articles were included. Compared with higher tidal volumes with FiO_2_-guided lower positive end-expiratory pressure [PEEP], the hazard ratios (HRs) for mortality were 0.624 (95% confidence interval (CI) 0.419–0.98) for lower tidal volumes with FiO_2_-guided lower PEEP and prone positioning and 0.572 (0.34–0.968) for pressure-controlled ventilation with FiO_2_-guided lower PEEP. Lower tidal volumes with FiO_2_-guided higher PEEP and prone positioning had the greatest potential to reduce mortality, and the possibility of receiving the first ranking was 61.6%. Permissive hypercapnia, recruitment maneuver, and low airway pressures were most likely to be the worst in terms of all-cause mortality. Compared with higher tidal volumes with FiO_2_-guided lower PEEP, pressure-controlled ventilation with FiO_2_-guided lower PEEP and lower tidal volumes with FiO_2_-guided lower PEEP and prone positioning ventilation are associated with lower mortality in ARDS patients. Lower tidal volumes with FiO_2_-guided higher PEEP and prone positioning ventilation and lower tidal volumes with pressure-volume (P–V) static curve-guided individual PEEP are potential optimal strategies for ARDS patients.

Acute respiratory distress syndrome (ARDS) is a common clinical condition with an incidence rate of nearly 9% in the intensive care unit (ICU)^1^. The mortality of ARDS is relatively high, at approximately 27–45%^2^. In the United States, there are an estimated 190,600 cases annually, resulting in 74,500 deaths and 3.6 million hospital days^3^. Mechanical ventilation is the most effective life-saving technique and can save a patient’s life by maintaining adequate tissue oxygenation^4^. However, the same ventilation interventions have exhibited different effects on mortality concerning ARDS in different clinical trials, and the issue remains controversial^4^,^5^,^6^,^7^,^8^,^9^. To compare the different ventilation strategies in the management of ARDS, many studies have attempted to identify optimal strategies for mechanical ventilation^10^,^11^. Traditional pairwise meta-analysis performs a systematic review and evaluation of different ventilator parameters, such as positive end-expiratory pressure (PEEP), tidal volume (VT) ventilation, prone positioning, and other parameters^12^,^13^,^14^,^15^,^16^. However, a standard pairwise meta-analysis can only compare two treatments (or classes) that have been directly compared in head to head trials^17^. The mechanical ventilation strategies for ARDS patients, however, include many ventilation parameters, such as PEEP, VT, recruitment maneuvers (RM), position, and others. Traditional pairwise meta-analysis can only compare a specific parameter between ventilation strategies and is unable to compare the entire set of parameters of different ventilation strategies; therefore, the ability to draw definitive conclusions from the results is limited. Network meta-analysis (also called multiple or mixed treatment comparison meta-analysis, MTC) permits the evaluation of the comparative effectiveness of multiple interventions, even though some pairs may not have been directly compared, and has the potential to reduce the uncertainty in treatment effect estimates^18^,^19^. By taking advantage of MTC, this study compared the effectiveness and safety of mechanical ventilation strategies with different parameters as follows: different ventilation modes; same ventilation mode with different parameter settings; same ventilation mode and same parameter settings with different parameter value; and same ventilation mode and same parameter settings with different operational techniques. We attempted to identify the optimal mechanical ventilation strategies for ARDS.

## Methods

We conducted our systematic review in accordance with the methods recommended in the PRISMA guidelines.

### Literature Search

RCTs were identified through electronic and manual searches. We searched the Cochrane Central Register of Controlled Trials (CENTRAL) in The Cochrane Library, EMBASE, MEDLINE, CINAHL, and the Web of Science using a combination of MeSH and text words (Appendix 1). We did not restrict our search based on language or year of publication. The last search update was in December 2015. We reviewed the reference lists of published meta-analyses. In addition, we manually searched the Index Medicus of RCTs, meta-analyses, and systematic reviews for studies that were missed in the initial electronic search.

### Inclusion and Exclusion Criteria

Two groups conducted the literature inclusion and exclusion process separately. When there was a discrepancy between the two groups, the selection committee met to reach an agreement on the inclusion and exclusion of the disputed literature. We first excluded the following literature: review studies, retrospective studies, observational studies, case reports, animal studies, studies conducted on children, studies regarding psychological mechanisms only, unrelated studies (such as studies of mechanical ventilation in patients with non-acute respiratory distress syndrome, or studies using other non-mechanical ventilation treatment strategies, such as medication for ARDS patients), duplicate reports, literature involving repeated experiments (commentary papers on specific studies or secondary analysis on experimental data), non-invasive mechanical ventilation studies, nonrandomized trials, and studies focused on comparing the effect of treatment before and after the application of ventilation intervention. Ultimately, randomized controlled trials on mechanical ventilation in adult ARDS patients were included. According to the modified Jadad scale^20^, all included studies were of relative high quality with a low bias risk (Table 1). No studies were excluded because of quality problems.

### Outcome Measures and Data Extraction

The extracted data included basic study information such as experimental design, experimental time, country of the study, the inclusion criteria, the age and gender of the included patients, detailed experimental procedure, specific parameter settings of the mechanical ventilation, clinical outcome, and safety outcomes of the patients. The primary outcome of this study regards the all-cause mortality of ARDS patients. If there were multiple all-cause mortalities calculated in selected studies, the mortality from the most long-term follow-up was extracted for analysis. The secondary outcome of this study regards barotrauma, duration of mechanical ventilation, ICU stay duration, and hospital stay duration. Two groups extracted the data separately; data comparison and verification were performed afterwards. If necessary, the extracted table was sent to the paper’s corresponding authors for supplementary data or verification. We also contacted corresponding authors to seek assistance in cases of missing data.

### Statistical Analysis

Multiple-treatment meta-analysis or network meta-analysis combines direct and indirect evidence for all relative treatment effects and provides estimates with maximum power^21^,^22^,^23^,^24^. Multiple-treatment meta-analysis was performed using the GeMTC R package^21^. As mortality was calculated across different time periods in the majority of the included studies, to maximize accuracy and effectiveness^22^,^25^, this study used hazard ratios (HRs) and 95% confidence intervals (CIs) to assess mortality in ARDS patients receiving mechanical ventilation. The statistical analysis was based on Poisson likelihoods with a log link function. We also used the odds ratio (OR) and 95% CIs to assess the incidence of barotrauma.

The statistical analysis was also based on binomial likelihoods with a logit link function. The CI was calculated with statistical methods based on Bayesian probability theory. The CI was considered statistically significant when the CI did not include 1.0. We used a random-effects model within a Bayesian framework using a Markov Chain Monte Carlo simulation to calculate HRs (mortality), ORs (barotrauma), and CI^24^. The models were run for 150,000 iterations, and convergence was assessed using the Brooks-Gelman-Rubin diagnostic^26^.

We used a technique known as ‘back-calculation’^23^ to evaluate the consistency of the network meta-analysis findings from direct versus indirect evidence. During this process, three types of model are estimated: unrelated study effects, unrelated mean effects, and consistency. The output of the summary function can be plotted for a visual representation. We used visual inspection of the forest plots and the I^2^ statistic to investigate the possibility of statistical heterogeneity and inconsistency between the direct and indirect effect estimates using the Higgins–Thompson method^27^ (low heterogeneity 25%, moderate 50%, and high 75%).

We also ranked the different interventions in terms of their likelihood of leading to the best results for each outcome. In the Markov chain Monte Carlo cycle, for each of the iterations, regimens were ranked according to the estimated log HR. The probability of a regimen being superior was then defined as the proportion of times a regimen ranked first. Each ventilation strategy was ranked by the estimated effect size. These probabilities sum to 1 for each treatment and each rank. A value of x% means that the strategy achieves x% effectiveness, and thus larger percentages denote more effective interventions. However, this denotation only represents one possibility without certainty.

### Sensitivity Analysis

We performed two sensitivity analyses, including and excluding specific studies that utilized substantially different study designs and populations. 1) According to Lopez’s trial^28^, age was independently associated with hospital outcome. In Bollen and co-workers’study^29^, the mean ages in the high-frequency oscillatory ventilation (HFOV) and traditional ventilation (CV) groups were 81.0 ± 20.5 and 81.7 ± 12.5 years, respectively, which were significantly different from those of the other groups. Age differences can have significant effects on mortality. We performed comparative studies before and after the exclusion of the study^29^. 2) Sensitivity analyses were performed on the studies’ follow-up times; two studies^30^,^31^ were eliminated due to follow-up times greater than 6 months.

## Results

**Figure 1:**
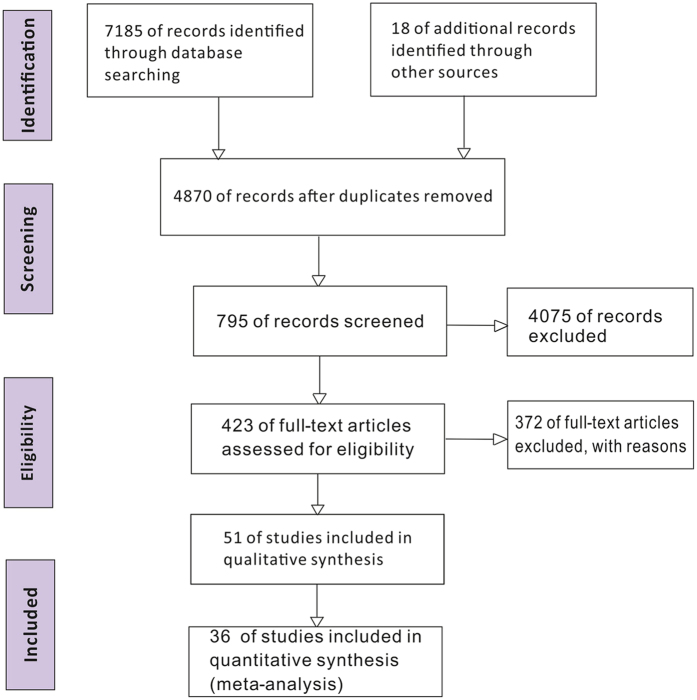
Flow diagram of the literature search.

**Figure 2:**
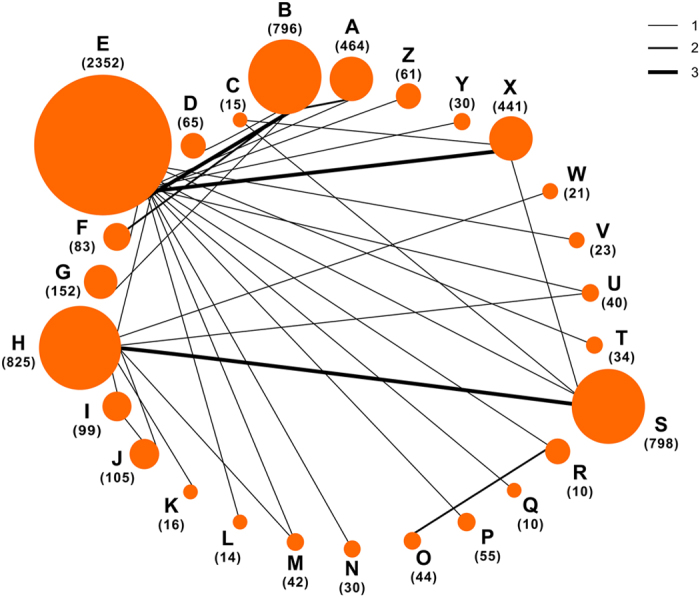
Network of the comparisons for the Bayesian network meta-analysis. The size of the nodes is proportional to the number of patients (in parentheses) randomized to receive the treatment. The width of the lines is proportional to the number of trials (beside the line) comparing the connected treatments.

We identified 7,185 studies by reviewing titles and abstracts (Fig. 1). After the initial screening, we retrieved the full texts of potentially eligible articles for detailed assessment. Thirty-six randomized controlled trials were included for meta-analysis (Table 2), with a total of 6,685 patients randomized to receive one of the 26 ventilation strategies (Fig. 2, Table 1). Compared with traditional meta-analysis, we sub-divided the ventilation strategies into 26 ventilation strategies: different ventilation modes; same ventilation mode with different parameter settings; same ventilation mode and same parameter settings with different parameter value; and same ventilation mode and same parameter settings with different operational techniques^12^,^32^,^33^.

**Figure 3:**
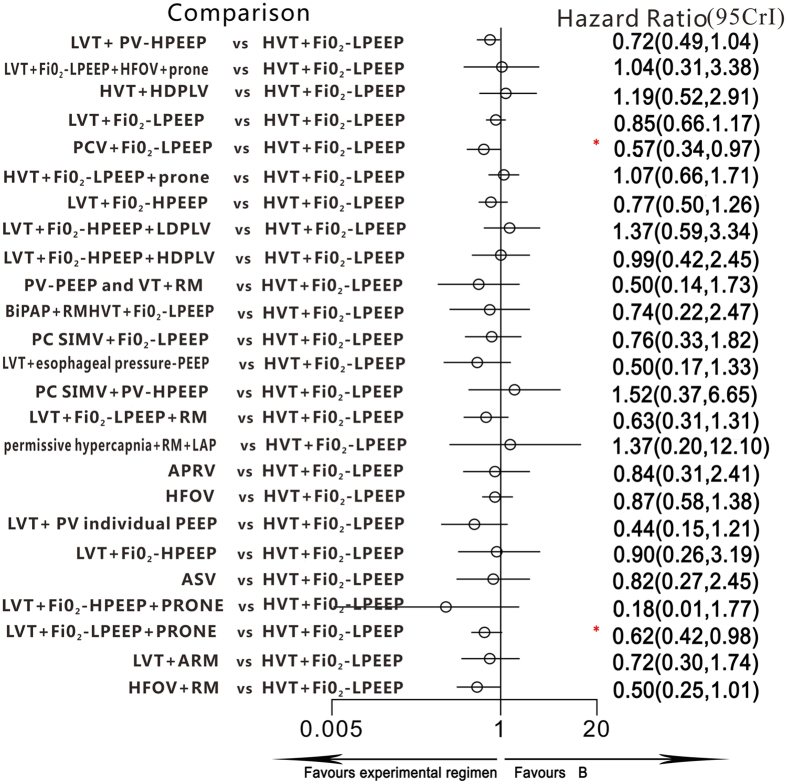
Hazard ratios for death in the Bayesian network meta-analysis versus B. CI = credible interval for Bayesian network meta-analysis. Hazard ratios (HRs) estimated from random effects, Bayesian network meta-analysis. ^*^95% CI does not contain 1.

All 36 trials reported information on all-cause mortality and were included for meta-analysis. Two trials were three-arm randomized studies, and the remaining trials were two-arm randomized studies. Compared with the ventilation strategy HVT + FiO_2_-LPEEP (higher tidal volumes with FiO_2_-guided lower PEEP), LVT + FiO_2_-LPEEP + PRONE (lower tidal volumes with FiO_2_-guided lower PEEP and prone positioning) and PCV + FiO_2_-LPEEP (pressure-controlled ventilation with FiO_2_-guided lower PEEP) were associated with lower mortality: the HRs and 95% CIs were 0.62 (0.42–0.98) and 0.57 (0.34–0.97) (Fig. 3), respectively. In addition, the HR and 95% CI between LVT + FiO_2_-LPEEP (lower tidal volumes with FiO_2_-guided lower PEEP) and LVT + FiO_2_-LPEEP + PRONE (lower tidal volumes with FiO_2_-guided lower PEEP and prone positioning) was 0.73 (0.53–1), although this was not statistically significant. All HR and 95% CIs of the ventilation strategies are shown in Table 3.

Most of the comparisons showed little or no heterogeneity. The endpoint of the I^2^ value of all-cause mortality exceeded 50% (I^2^ = 51.8%) in only one of the comparisons, ventilation strategy HFOV (high-frequency oscillatory ventilation) vs. ventilation strategy LVT + FiO_2_-HPEEP (lower tidal volumes with FiO_2_-guided higher PEEP), indicating the presence of moderate heterogeneity. Comparing the all-cause mortality results from traditional pairwise meta-analysis and network meta-analysis did not suggest any inconsistency between direct and indirect evidence (Appendix 3). The sensitivity analyses regarding age and follow-up period did not affect the results of the meta-analyses on mortality in ARDS patients. After the exclusion of three studies^29^,^30^,^31^, the ventilation strategies PCV + FiO_2_-LPEEP (pressure controlled ventilation with FiO_2_-guided lower PEEP) and LVT + FiO_2_-LPEEP + PRONE (lower tidal volumes with FiO_2_-guided lower PEEP and prone positioning) still had statistically significant reductions in mortality in ARDS patients compared with HVT + FiO_2_-LPEEP (higher tidal volumes with FiO_2_-guided lower PEEP).

Twenty-two included studies reported the incidence of barotrauma as a secondary outcome. Barotrauma was involved in 15 ventilation strategies. Using OR as the “combining effect size,” ventilation strategy PV-PEEP and VT + RM (static pressure-volume [P–V] curve was measured daily, PEEP and VT were set based on the P–V variation, and the open-lung potential was evaluated before recruitment maneuvers) was associated with a lower incidence of barotrauma compared to other ventilation strategies. The ORs and 95% CIs of different types of ventilation strategies are shown in Table 3. Twelve studies^4^,^7^,^29^,^30^,^34^,^35^,^36^,^37^,^38^,^39^,^40^,^41^ reported the lengths of mechanical ventilation, 14^7^,^31^,^36^,^38^,^39^,^40^,^41^,^42^,^43^,^44^,^45^,^46^,^47^,^48^ reported ICU stay durations, and 8^38^,^40^,^41^,^42^,^44^,^45^,^46^,^47^ reported hospital stay durations. Unfortunately, certain treatment strategies in these studies were isolated and distinctive from other treatment strategies and thus cannot be included in network-meta-analysis.

**Figure 4:**
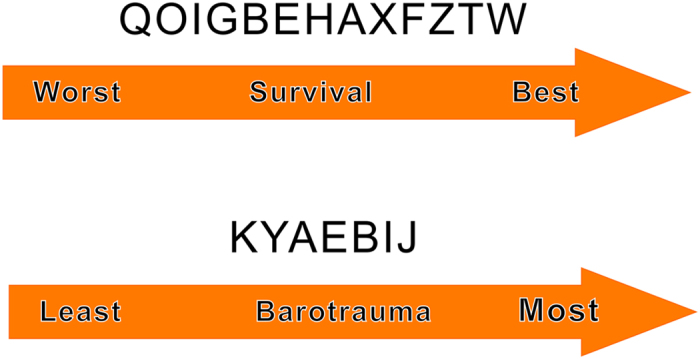
Ranking of treatments in terms of all-cause mortality benefit and incidence of barotraumas. The probability ranking represents only a possibility without certainty; combining the direct and indirect evidence analysis on overall mortality has more reference significance.

In Fig. 4, we summarize the rankings of the different competing treatment strategies in terms of all-cause mortality and the incidence of barotrauma, with details provided in Appendices 4 and 5. Ventilation strategy LVT + FiO_2_-HPEEP + PRONE (lower tidal volumes with FiO_2_-guided higher PEEP and prone positioning) had the greatest potential to reduce mortality, and the possibility of its receiving the first ranking was 61.6%. The second ranking was ventilation strategy LVT + PV individual PEEP (lower tidal volumes with P–V static curve-guided individual PEEP), with a possibility of 18.4%. Permissive hypercapnia + RM + LAP (permissive hypercapnia, recruitment maneuvers, and low airway pressures) was the worst in terms of all-cause mortality. In terms of reducing the incidence of barotrauma, ventilation strategy PV-PEEP and VT + RM (static pressure-volume [P–V] curve was measured daily, PEEP and VT were set based on P–V variation, and the open-lung potential was evaluated before recruitment maneuvers) was ranked highest, with a possibility of 63.4%. Ventilation strategy LVT + FiO_2_-HPEEP + HDPLV (lower tidal volumes with FiO_2_-guided higher PEEP and higher-dose partial liquid ventilation) had the highest probability of causing barotrauma.

## Discussion

A complete ventilation strategy for ARDS includes various different respiratory parameters. However, because of its limitations, conventional pair-wise meta-analysis can only compare two different parameters, or different values of one specific parameter, but cannot be used to compare two complete ventilation strategies. Therefore, only one ventilatory parameter can be used as the primary study variable, and all other ventilatory parameters can only be considered as background variables. However, clinical studies have shown that other ventilatory parameters might also affect the outcome of ARDS patients^4^,^46^. Santa Cruz R and colleagues^12^ included 7 studies in a meta-analysis study on the effect of higher-PEEP and lower-PEEP on the mortality and barotrauma incidence of ARDS patients. In their study, PEEP value was the only comparative variable. Other respiratory parameters, such as the PEEP setting mode and tidal volume, were not considered. For example, in the original 3 studies^32^,^39^,^49^, the PEEP values, tidal volumes and PEEP setting modes were all set differently. In the study^49^, the ventilation mode of higher tidal volumes with FiO_2_-guided PEEP was set in the control group; the ventilation mode of P–V-guided lower PEEP was set in the experimental group. The tidal volume was consistently set lower in both of the studies^32^,^39^. Similarly, Hodgson and co-workers^50^ conducted a conventional meta-analysis to study the effect of recruitment maneuvers on mild ARDS patients. The included studies by Amato^49^, Brower^51^, and Meade^52^ had different higher or lower initial tidal volume settings and different PEEP setting modes; however, these two different parameter settings were ignored in Hodgson C’s study. Some studies^4^ have shown that a difference in tidal volume can also produce different mortality in ARDS patients. Lower tidal volume ventilation could reduce mortality in ARDS patients. Studies^46^ have also indicated that the PEEP setting mode has different effects on ARDS patients compared with the FiO_2_-guided group. Multiple-organ-dysfunction-free days, respiratory-failure-free days, and hemodynamic-failure-free days at 28 days were significantly lower in subjects with compliance-guided PEEP settings. Conventional meta-analysis cannot simultaneously study various ventilation parameters as a complete treatment strategy due to its methodological limitations; therefore, the study results have limited reference significance. The greatest difference between our study and conventional meta-analysis is that this study not only examined individual parameters but also simultaneously examined various parameters as a complete treatment strategy. Our method is more reasonable, more scientific, and able to provide a more direct reference standard for clinical practitioners.

The results of this meta-analysis showed that compared with the ventilation strategy (higher tidal volumes with FiO_2_-guided lower PEEP), both (lower tidal volumes with FiO_2_-guided lower PEEP and prone positioning) and (pressure-controlled ventilation with FiO_2_-guided lower PEEP) were associated with lower mortality in patients, and the difference was statistically significant.

When summarizing the possible rankings of the different ventilation strategies on ARDS patients’ mortality, we found that (lower tidal volumes with FiO_2_-guided higher PEEP and prone positioning) was the optimal ventilation strategy, and (lower tidal volumes with P–V static curve guided individual PEEP) ranked second. In addition, (permissive hypercapnia, recruitment maneuvers, and low airway pressures) had the highest potential mortality among all ventilation strategies.

The major cause of death in ARDS is multiple organ failure resulting from systemic inflammatory mediator release^53^. Ventilator-associated pneumonia (VAP) may contribute to the mortality associated with ARDS^54^. Lung volume was significantly reduced in patients with ARDS. The number of alveoli participating in normal ventilation function, which is referred to as “baby lung,” was also reduced^55^. Conventional tidal volume can lead to increased tension in the walls of alveoli or stress in the alveoli^56^,^57^. In comparison, lower tidal volume can avoid the overexpansion of residual normal alveoli, alleviate lung injury, and reduce the release and spread of inflammatory mediators^58^, which can improve tissue oxygenation and simultaneously significantly reduce the incidence of ventilator-associated lung injury^49^,^59^. By reducing the overexpansion of alveoli, improving ventilation-perfusion matching (V/Q) and lung mechanics^60^, promoting lung recruitment^61^, and improving the excretion of airway secretions^62^, prone positioning can simultaneously improve tissue oxygenation^6^ and reduce the incidence of VAP^63^. Pressure-controlled ventilation can also produce relatively good physiological effects, such as increased static lung compliance, reduced mechanical ventilation time^64^, and improved tissue oxygenation^65^. The above theories might be potential mechanisms through which (lower tidal volumes with FiO_2_-guided lower PEEP and prone positioning) and (pressure-controlled ventilation with FiO_2_-guided lower PEEP) are associated with lower mortality. Because the study sample sizes of (lower tidal volumes with FiO_2_-guided higher PEEP and prone positioning) and (lower tidal volumes with P–V static curve-guided individual PEEP) were relatively small, the results were more likely to have bias^66^. Therefore, regarding possible ranking, combining the direct and indirect evidence analysis on overall mortality has more reference significance. In our study, we divided ventilation strategies into groups in detail, avoiding the limit effect of single parameters, and we integrated the combined actions of different ventilation parameters or same ventilation modes with different parameter settings, which can more comprehensively account for the effectiveness of the entire mechanical ventilation strategy.

(Permissive hypercapnia, recruitment maneuvers and low airway pressures) has the highest potential mortality among all ventilation strategies. During RM, it can increase the resistance of lung vessels and transiently decrease cardiac output and mean arterial pressure^67^,^68^. It can also decrease tissue oxygen saturation^51^,^69^, causing harmful hemodynamic effects. The death of the patient can also be caused by RM-induced complications, such as hemodynamic compromise or pneumothorax. Therefore, (permissive hypercapnia, recruitment maneuvers and low airway pressures) is the ventilation strategy with the highest potential mortality.

In comparing different models of mechanical ventilation-induced barotrauma incidence, there is significant inconsistency between the direct and indirect evidence (Appendix 6). After carefully analyzing the incidence data of mechanical ventilation-induced barotrauma across all mechanical ventilatory strategies, we found that a ventilation strategy can have significantly different incidences of barotrauma in different experiments. Among them, the variability of (higher tidal volumes with FiO_2_-guided lower PEEP) in the incidence of barotrauma was the largest, with a range from 3.8% to 41.6%^8^,^49^. After careful comparison of the included studies, we found that the inclusion criteria for barotrauma was not exactly the same. For example, barotrauma was defined in the study as any new pneumothorax, pneumomediastinum, subcutaneous emphysema, or pneumatocele with a diameter of more than 2 cm after randomization^32^. Barotrauma has also been defined as including pneumothorax, pneumomediastinum, pneumoperitoneum, pneumopericardium, or subcutaneous emphysema^42^. However, other studies^10^,^34^,^35^ have recorded the incidence of barotrauma as including only pneumothorax. In addition, we speculate that due to the different limitations of medical conditions at the experimental sites and the difference in diagnostic experience of the clinical practitioners, certain barotrauma might not be detected, leading to relatively the large difference in the incidence of barotrauma in different clinical experiments. These phenomena are the most likely causes of inconsistencies between analysis from the direct and indirect evidence, and these inconsistencies mean the network meta-analysis of the incidence of barotrauma in ARDS patients might have very limited reference value. Therefore, we suggest that the inclusion of barotrauma should be performed according to identical diagnostic procedures and diagnostic standards in future studies, to obtain more unified clinical data and assist in further comparison analysis.

### Limitations

As an innovative study, this study also has certain limitations. 1) The included trials numbers of (lower tidal volumes with FiO_2_-guided higher PEEP and prone positioning), (lower tidal volumes with P–V static curve-guided individual PEEP), and (permissive hypercapnia, recruitment maneuvers and low airway pressure) ventilation were limited. In addition, there are few direct head-to-head comparisons of related treatment strategies. Therefore, the results can easily exhibit deviation. Future large sample size, multi-center, parallel-group and direct comparison studies are needed to validate the results of our study. For example, in the original studies on mechanical ventilation, Villar^70^ and Mercat^10^ studied LV_T_ + PV-HPEEP vs. HVT + FiO_2_-LPEEP and LV_T_ + PV-HPEEP vs. LVT + FiO_2_-LPEEP, respectively. Based on transitivity, we could obtain comparison results of HVT + FiO_2_-LPEEP and LVT + FiO_2_-LPEEP; however, we could not establish a relationship between LVT + FiO_2_-HPEEP and LV_T_ + PV-HPEEP, and we could not obtain comparison results of LV_T_ + PV-HPEEP and LVT + FiO_2_-HPEEP by network meta-analysis. 2) Because the original experimental results are not complete, no network meta-analysis was conducted on the time of mechanical ventilation, ICU stay duration, and hospital stay duration. Therefore, the results of this study are relatively simple and lack a comprehensive conclusion.

## Conclusion

We conducted a network meta-analysis based on direct and indirect evidence to compare the currently applied invasive mechanical ventilation strategies with respect to all-cause mortality in ARDS patients. The results indicated that the ventilation strategies (higher tidal volumes with FiO_2_-guided lower PEEP), (pressure-controlled ventilation with FiO_2_-guided lower PEEP) and (lower tidal volumes with FiO_2_-guided lower PEEP and prone positioning) were associated with lower mortality in ARDS patients. Ventilation strategies W (lower tidal volumes with FiO2-guided higher PEEP and prone positioning) and T (lower tidal volumes with P–V static curve-guided individual PEEP) are potential optimal ventilation strategies for ARDS patients.

### Key messages

Mechanical ventilation is the most effective life-saving technique and can save an ARDS patient’s life.

We attempted to identify optimal strategies for mechanical ventilation of patients with ARDS by taking advantage of a network meta-analysis.

Ventilation strategies (higher tidal volumes with FiO_2_-guided lower PEEP, pressure-controlled ventilation with FiO_2_-guided lower PEEP and lower tidal volumes with FiO_2_-guided lower PEEP and prone positioning) were associated with lower mortality in ARDS patients.

The ventilation strategies LVT + FiO_2_-HPEEP + PRONE (lower tidal volumes with FiO_2_-guided higher PEEP and prone positioning) and LVT + PV individual PEEP (lower tidal volumes with P–V static curve-guided individual PEEP) are potential optimal ventilation strategies for ARDS patients.

## Additional Information

**How to cite this article**: Wang, C. *et al.* Lung ventilation strategies for acute respiratory distress syndrome: a systematic review and network meta-analysis. *Sci. Rep.* **6**, 22855; doi: 10.1038/srep22855 (2016).

## Supplementary Material

Supplementary Information

## Figures and Tables

**Table 1:** Twenty-six ventilation strategies for ARDS.

A	LVT + PV-HPEEP	lower tidal volumes with P-V static curve-guided higher PEEP
B	HVT + FiO_2_-LPEEP	higher tidal volumes with FiO_2_-guided lower PEEP
C	LVT + FiO_2_-LPEEP + HFOV + prone	lower tidal volumes with FiO_2_-guided lower PEEP and high-frequency oscillatory ventilation following prone positioning
D	HVT + HDPLV	higher tidal volumes with higher dose partial liquid ventilation
E	LVT + FiO_2_-LPEEP	lower tidal volumes with FiO_2_-guided lower PEEP
F	PCV + FiO_2_-LPEEP	pressure controlled ventilation with FiO_2_-guided lower PEEP
G	HVT + FiO_2_-LPEEP + prone	higher tidal volumes with FiO_2_-guided lower PEEP and prone positioning
H	LVT + FiO_2_-HPEEP	lower tidal volumes with FiO_2_-guided higher PEEP
I	LVT + FiO_2_-HPEEP + LDPLV	lower tidal volumes with FiO_2_-guided higher PEEP and lower dose partial liquid ventilation
J	LVT + FiO_2_-HPEEP + HDPLV	lower tidal volumes with FiO_2_-guided higher PEEP and higher dose partial liquid ventilation
K	PV-PEEP and VT + RM	static pressure-volume (P-V) curve was measured daily, PEEP and VT were set based on P-V variation. and the open-lung potential was evaluated before recruitment maneuvers
L	BiPAP + RM	BiPAP mechanical ventilation combined with lung recruitment maneuvers
M	PC SIMV + FiO_2_-LPEEP	pressure-controlled SIMV (synchronized intermittent ventilation)-mode with pressure support and FiO_2_-guided lower PEEP
N	LVT + esophageal pressure- PEEP	lower tidal volumes with esophageal-pressure guided PEEP
O	PC SIMV + PV-HPEEP	pressure-controlled SIMV (synchronized intermittent ventilation)-mode with pressure support and P-V static curve-guided higher PEEP
P	LVT + FiO_2_-LPEEP + RM	lower tidal volumes with FiO_2_-guided lower PEEP and lung recruitment maneuvers
Q	permissive hypercapnia + RM + LAP	permissive hypercapnia, recruitment maneuvers and low airway pressures
R	APRV	airway pressure release ventilation
S	HFOV	high-frequency oscillatory ventilation
T	LVT + PV individual PEEP	lower tidal volumes with P-V static curve-guided individual PEEP
U	LVT + FiO_2_-HPEEP	lower tidal volumes with FiO_2_-guided higher PEEP and extracorporeal CO2 elimination
V	ASV	Adaptive support ventilation
W	LVT + FiO_2_-HPEEP + PRONE	lower tidal volumes with FiO_2_-guided higher PEEP and prone positioning
X	LVT + FiO_2_-LPEEP + PRONE	lower tidal volumes with FiO_2_-guided lower PEEP and prone positioning
Y	LVT + ARM	lower tidal volumes with PEEP titration after an alveolar recruitment maneuver (ARM)
Z	HFOV + RM	high-frequency oscillatory ventilation with tracheal gas insufflation and recruitment maneuver

Abbreviations: ARDS, Acute Respiratory Distress Syndrome; PEEP, Positive End-expiratory Pressure.

**Table 2:** Characteristics of randomized controlled trials of twenty-six ventilation strategies for ARDS.

Source	Ventilation strategies	Jadadscale	No. of Patients	Age(y)Mean ± SD	Oxygenation index(mmHg) Mean ± SD	Diagnosis	Result				
							Mortality	Length ofmechanical ventilation (d) Mean ± SD	ICU length of stay (d) Mean ± SD	Barotrauma(n)	Hospital Lengthof stay (d)Mean ± SD
Amato*et al.*^49^	HVT + FiO_2_−LPEEP vs. HVT + FiO_2_−LPEEP	6	53	33 ± 13/36 ± 14	112/134^a^	ARDS	*Death In hospital	Not reported	Not reported	2/10	Not reported
							Death in the ICU				
							Mortality at 28 days				
Brochard*et al.*^7^	LVT + FiO_2_−LPEEP vs. HVT + FiO_2_−LPEEP	6	116	57.0 ± 15.3/56.5 ± 15.3	144/155^a^	ARDS	*Mortality at 60 days	23.1 ± 20.2/21.4 ± 16.3	33.5 ± 28.7/29.7 ± 19.4	Not reported	Not reported
Confalonieri*et al.*^8^	LVT + FiO_2_−LPEEP vs. HVT + FiO_2_−LPEEP	6	52	49.8/46.9^a^	150 ± 69/128 ± 51	ARDS	*Death In hospital	Not reported	Not reported	2/1	Not reported
Esteban*et al.*^42^	LVT + FiO_2_−LPEEP vs. PCV + FiO_2_−LPEEP	7	79	59 ± 16/56 ± 17	131 ± 48/126 ± 47	ARDS	*Death In hospital	Not reported	25 ± 19/21 ± 15	4/6	30 ± 24/27 ± 20
							Death in the ICU				
ARDSnetwork^4^	LVT + FiO_2_−LPEEP vs. HVT + FiO_2_−LPEEP	6	861	51 ± 17/52 ± 18	138 ± 64/134 ± 58	ARDS	*Death In hospital	12 ± 11/10 ± 11	Not reported	43/47	Not reported
Gattinoni^6^	HVT + FiO_2_−LPEEP vs. HVT + FiO_2_−LPEEP + prone	5	304	57 ± 16/59 ± 17	129.5 ± 47.5/125.3 ± 48.8	ARDS	*Mortality at 10 days	Not reported	Not reported	Not reported	Not reported
Kacmarek*et al.*^35^	LVT + FiO_2_−HPEEP vs. LVT + FiO_2_−HPEEP + LDPLV vs. LVT + FiO_2_−HPEEP + HDPLV	7	311	46 ± 12/45 ± 14/45 ± 13	147 ± 54/137 ± 58/143 ± 52	ARDS	*Mortality at 28 days	13.0 ± 9.3/7.4 ± 8.5/9.9 ± 9.1	Not reported	3/8/13	Not reported
Long*et al.*^36^	LVT + FiO_2_−HPEEP vs. PV−PEEP and VT + RM	4	30	61 ± 16/58 ± 18	142 ± 34/120 ± 29	ARDS	*Mortality at 28 days	4(1–8)/11(5–8)^b^	3(0–8)/ 11(5–16)^b^	2/0	Not reported
Wang*et al.*^37^	BiPAP + RM vs. LVT + FiO_2_−LPEEP	4	28	36 ± 8/38 ± 9	180 ± 10/179 ± 9	ARDS	*Mortality at 28 days	14 ± 3/19 ± 3	Not reported	1/1	Not reported
Didier*et al.*^71^	LVT + FiO_2_−LPEEP + PRONEvs. HFOV vs. LVT + FiO_2_−LPEEP + HFOV + prone	4	43	52 ± 13/45 ± 14/56 ± 17	122 ± 28	ARDS	*Death in the ICU	Not reported	Not reported	Not reported	Not reported
Xi*et al.*^44^	LVT + FiO_2_−LPEEP + RM vs. LVT + FiO_2_−LPEEP	6	110	62.2 ± 16.0/65.5 ± 15.2	93.8 (68.7–150.0)/120.0 (88.3–140.0)^b^	ARDS	*Death in hospital	10.8 ± 10.1/10.8 ± 10.1	22.5 ± 22.2/19.8 ± 24.8	Not reported	43.2 ± 45.6/33.2 ± 34.0
							Mortality at 28 days				
							Death in ICU				
Hodgson^40^	permissive hypercapnia + RM + LAP vs. LVT + FiO_2_−LPEEP	7	20	60 ± 5/58 ± 4	155/149^a^	ARDS	*Death in hospital	180(87–298)/341(131–351)^b^	9.9 (5.6–14.8)/16.0 (8.1–19.3)^b^	Not reported	17.9 (13.7–34.5)/24.7 (20.5–39.8)^b^
Dolinay*et al.*^72^	APRV vs. LVT + FiO_2_−LPEEP	4	34	Not reported	Not reported	ARDS	*Death in hospital	6.4/7.7^a^	Not reported	Not reported	8.6/10.3^a^
							Death in ICU				
Young*et al.*^45^	HFOV vs. LVT + FiO_2_−LPEEP	5	795	55.4 ± 16.2	113 ± 38/113 ± 37	ARDS	Mortality at 30 days	Not reported	17.6 ± 16.6/16.1 ± 15.2	Not reported	33.9 ± 41.6/33.1 ± 44.3
							Death in ICU				
							*Death in hospital				
Ferguson*et al.*^73^	HFOV vs. LVT + FiO_2_−HPEEP	5	548	55 ± 16/54 ± 16	121 ± 46/114 ± 38	ARDS	*Death in hospital	11(7–19)/10(6–18)^b^	Not reported	46/34	30(16–45)/25(15–41)^b^
							Death in ICU				
							Mortality at 28 days				
Pintado*et al.*^46^	LVT + PV individual PEEP vs. LVT + FiO_2_−LPEEP	5	70	55.6 ± 3.1	133.15 ± 5.88/146.33 ± 6.19	ARDS	*Mortality at 28 days	Not reported	21 (15–46)/20 (12–29)^b^	6/6	55 ± 7/32 ± 3
Bein*et al.*^47^	LVT + FiO_2_−HPEEP vs. LVT + FiO_2_−HPEEP	5	79	49.8 ± 12/48.7 ± 17	152 ± 37/168 ± 37	ARDS	*Death in hospital	Not reported	31.3 ± 23/22.9 ± 11	Not reported	46.7 ± 33/35.1 ± 17
Rappaport*et al.*^64^	HVT + FiO_2_−LPEEP vs. PCV + FiO_2_−LPEEP	6	27	51.6 ± 6.3/43.1 ± 4.3	77.5 ± 9.85/74.4 ± 6.54	ARDS	*Mortality at 30 days	Not reported	Not reported	1/0	Not reported
Hirschl*et al.*^34^	HVT + HDPLV vs. HVT + FiO_2_−LPEEP	6	90	44 ± 2/41 ± 3	178 ± 12/198 ± 22	ARDS	*Mortality at 28 days	6.3 ± 1/6.7 ± 1.8	Not reported	15/5	Not reported
Varpula *et al.*^74^	APRV vs. PC SIMV + PV-HPEEP	4	58	50.0 (38.5–60.5)/44.0(35.5–53.0)^b^	150.0 ± 10.5/164.3 ± 10.5	ARDS	Mortality at day 28	13.4 ± 1.7/12.2 ± 1.5	11.9 ± 1.7/10.7 ± 1.4	Not reported	Not reported
							*Mortality at 1 year				
Roy *et al.*^32^	LVT + FiO_2_−LPEEP vs. LVT + FiO_2_−HPEEP	7	549	49 ± 17/54 ± 17	165 ± 77/151 ± 67	ARDS	*Death in hospital	Not reported	Not reported	27/30	Not reported
CHEN *et al.*^70^	PCV + FiO_2_−LPEEP vs. HVT + FiO_2_-LPEEP	4	56	16–68/18–65^c^	< 200	ARDS	Not reported	Not reported	Not reported	2/8	Not reported
Taccone *et al.*^31^	LVT + FiO_2_−LPEEP + PRONE vs. LVT + FiO_2_−LPEEP	7	342	60/61^a^	141/77^a^	ARDS	Mortality at day 28	25(12–28)/19(9–28)^b^	17.5(9–31)/16(8–26)^b^	Not reported	Not reported
							Death in ICU				
							*Mortality at 6 months				
Varpula *et al.*^75^	APRV vs. PC SIMV + PV-HPEEP	4	37	Not reported	158/65^a^	ARDS	*Mortality at day 7	Not reported	Not reported	Not reported	Not reported
Agarwal *et al.*^41^	LVT + FiO_2_−LPEEP vs. ASV	7	48	29.7 ± 11.6/31.4 ± 14.9	96.6 ± 34.5/107.3 ± 41.9	ARDS	*Death in hospital	6 (3.5–11.5)/5(3–11)^b^	9 (4.5–15.5)/8(6–14)^b^	Not reported	11 (6.5–18.5)/11 (8–16)
Derdak^30^	HFOV vs. LVT + FiO_2_−HPEEP	7	148	48 ± 17/51 ± 18	114 ± 37/111 ± 42	ARDS	Mortality at 30 days	22 ± 21/20 ± 31	Not reported	7/9	Not reported
							*Mortality at 6 months				
Bollen^29^	HFOV vs. LVT + FiO_2_−HPEEP	7	61	81.0 ± 20.5/81.7 ± 12.5	< 200	ARDS	*Mortality at 30 days	20 ± 6/18 ± 5	Not reported	1/1	Not reported
Villar *et al.*^76^	LVT + PV-HPEEP vs. HVT + FiO_2_−LPEEP	7	95	48(28–62)/52(40–69)	124 ± 54/139 ± 43	ARDS	Death in ICU	10.9 ± 9.4/6.0 ± 7.9	Not reported	2/4	Not reported
							*Death in hospital				
Voggenreiter *et al.*^77^	LVT + FiO_2_−HPEEP + PRONE vs. LVT + FiO_2_−HPEEP	5		40 ± 14/43 ± 10	215 ± 63/228 ± 75	ARDS	*Mortality at 90 days	30 ± 17/33 ± 23	Not reported	Not reported	Not reported
Mercat *et al.*^10^	LVT + PV-HPEEP vs. LVT + FiO_2_−LPEEP	5	767	60 ± 16/60 ± 15	143 ± 57/144 ± 58	ARDS	*Death in hospital	7(0.0–19)/3 (0.0–17)^d^	Not reported	26/22	Not reported
							Mortality at 28 days				
							Mortality at 60 days				
Guérin *et al.*^5^	LVT + FiO_2_−LPEEP + PRONE vs. LVT + FiO_2_−LPEEP	5	466	58 ± 16/60 ± 16	100 ± 20/100 ± 30	ARDS	Mortality at 28 days	17 ± 16/19 ± 21	24 ± 22/26 ± 27	15/13	Not reported
							*Mortality at 90 days				
Fernandez *et al.*^38^	LVT + FiO_2_−LPEEP + PRONE vs. LVT + FiO_2_−LPEEP	5	40	53.9 ± 17.9/55.3 ± 14.6	157.8 ± 83.8/153.2 ± 59.4	ARDS	*Mortality at 60 days	11.9 ± 9.2/15.7 ± 16.9	14.7 ± 9.7/17.5 ± 16.1	0/1	31.3 ± 26.4/25.5 ± 17.4
Huh *et al.*^39^	LVT + ARM vs. LVT + FiO_2_−LPEEP	4	57	55.0 ± 3.7/62.0 ± 2.2	110.8 ± 6.3/ 115.0 ± 8.5	ARDS	*Mortality at 60 days	19.8 ± 0.5/15.2 ± 3.2	25.1 ± 5.6/21.4 ± 5.3	3/3	Not reported
							Mortality at 28 days				
							Death in ICU				
Mentzelopoulos*et al.*^78^	HFOV + RM vs. LVT + FiO_2_−LPEEP	7	125	50.7 ± 17.7/52.9 ± 17.1	96.5 ± 31.3/106.9 ± 27.7	ARDS	*Death in hospital	Not reported	31.9 ± 23.4/37.4 ± 19.6	Yes	52.8 ± 30.6/64.2 ± 27.8
Sun *et al.*^79^	PC SIMV + FiO_2_−LPEEP vs. LVT + FiO_2_−LPEEP	4	85	50 ± 17/51 ± 8	≤ 300	ARDS	*Mortality at 28 days	8.4 ± 2.1/10.7 ± 1.2	10.2 ± 2.2/13.7 ± 3.1	Not reported	Not reported
Talmor*et al.*^43^	LVT + esophageal pressure-PEEP vs. LVT + FiO_2_−LPEEP	6	61	54.5 ± 16.1/51.2 ± 23.0	147 ± 56/145 ± 57	ARDS	*Mortality at 180 days	12.0(7.0–27.5)/16.0(7.0–20.0)^d^	15.5(10.8–28.5)/13.0(7.0–22.0)^d^	0/0	Not reported
							Mortality at 28 days				

(Regimens are described in Table 3).

^*^primary outcomes for overall survival.

^a^mean.

^b^median (interquartile range).

^c^range.

^d^mean (interquartile range). Abbreviations: ARDS, Acute Respiratory Distress Syndrome; ICU, Intensive Care Unit. Reference to ARDS Clinical Trials Network (37), ARDS Network (4) and Taccone’s (31) method, based on the primary study, high PEEP was defined by PEEP < 10 cm H2O, low tidal volume was defined by VT < 8 mL/kg.

**Table 3:**
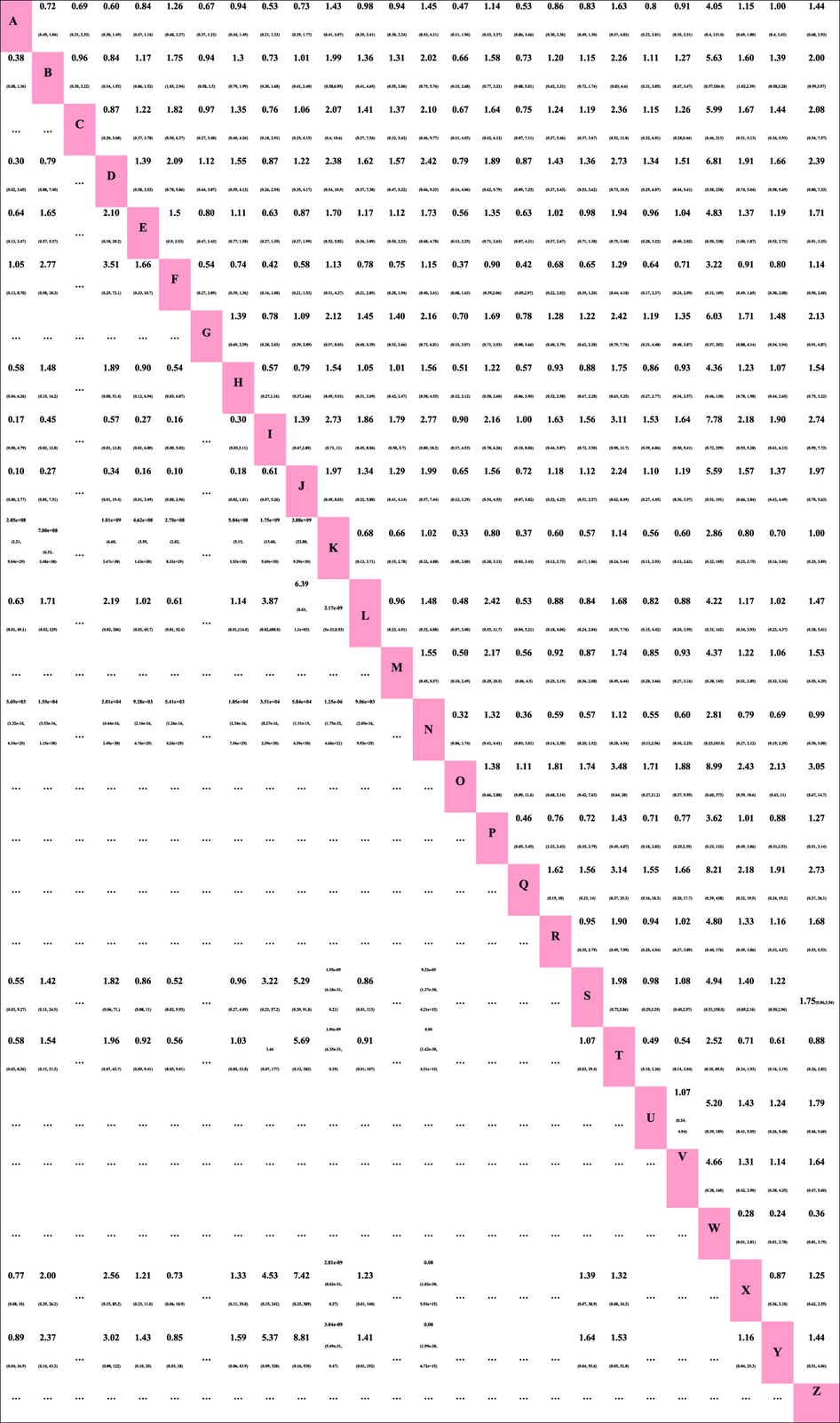
Pooled hazard ratios for death and pooled odds ratios for the incidence of barotraumas.

Hazard ratios for death are above the diagonal line (row defining treatment *vs*. column defining treatment), while odds ratios for the incidence of barotraumas are below the diagonal line (column defining treatment *vs*. row defining treatment). If the range of the 95% CI for HR and OR does not contain 1, the red numbers indicate corresponding values. … = not compared; CI = credible interval; HR = hazard ratio; OR = odds ratios.
