# Supplementary material for: Lung ventilation strategies for acute respiratory distress syndrome: a systematic review and network meta-analysis
Source: Sci Rep. 2016 Mar 9;6:22855. doi: 10.1038/srep22855 (PMC4783789; doi:10.1038/srep22855)
Supplement: Supplementary Information [file srep22855-s1.pdf]

Lung ventilation strategies for acute respiratory distress syndrome: a systematic review and network meta-analysis

Changsong Wang, Xiaoyang Wang, Chunjie Chi, Libo Guo, Lei Guo, Nana Zhao, Weiwei Wang, Xin Pi, Bo Sun, Ailing Lian, Jinghui Shi, Enyou Li

Appendix 1. Search Strategy

1. Search strategy for CENTRAL, The Cochrane Library

#1 MeSH descriptor: [Respiratory Distress Syndrome, Adult] explode all trees
#2 acute lung injury:ti,ab,kw (Word variations have been searched)
#3 Adult Respiratory Distress Syndrome:ti,ab,kw (Word variations have been searched)
#4 Acute Respiratory Distress Syndrome:ti,ab,kw (Word variations have been searched)
#5 ARDS or ALI:ti,ab,kw (Word variations have been searched)
#6 #1 or #2 or #3 or #4 or #5
#7 MeSH descriptor: [Respiration, Artificial] explode all trees
#8 lung protective ventilation strategy:ti,ab,kw (Word variations have been searched)
#9 protective near ventilation:ti,ab,kw (Word variations have been searched)
#10 mechanical ventilation:ti,ab,kw (Word variations have been searched)
#11 LPVS:ti,ab,kw (Word variations have been searched)
#12 #7 or #8 or #9 or #10 or #11
#13 #6 and #12

2. Search strategy for EMBASE (OvidSP)

1. respiratory distress syndrome/ or respiratory distress/ or acute lung injury/ or adult respiratory distress syndrome/ or acute respiratory failure/ or adult respiratory distress syndrome.mp. or acute lung injury.mp. or acute respiratory distress syndrome.mp. or (ards or ali).mp.
2. respiration, artificial/ or lung protective ventilation strategy/ or (protective adj3 ventilation).mp. or mechanical ventilation.mp. or LPVS.mp. [mp=title, abstract, subject headings, heading word, drug trade name, original title, device manufacturer, drug manufacturer, device trade name, keyword]
3. 1 and 2
4. (placebo.sh. or controlled study.ab. or random*.ti,ab. or trial*.ti,ab. or ((singl* or doubl* or trebl* or tripl*) adj3 (blind* or mask*)).ti,ab.) not (animals not (humans and animals)).sh.
5. 3 and 4

3. Search strategy for MEDLINE (OvidSP)

1. exp Respiratory Distress Syndrome, Adult/ or Adult Respiratory Distress Syndrome/ or Acute Lung Injury/ or Acute Respiratory Distress Syndrome/ or ARDS.mp. or ALI.mp.
2. exp Respiration, Artificial / or lung protective ventilation strategy / or (protective adj3 ventilation).mp. or mechanical ventilation or LPVS.mp.

3. 1 and 2

4. ((randomized controlled trial or controlled clinical trial).pt. or randomized.ab. or placebo.ab. or clinical trials as topic.sh. or randomly.ab. or trial.ti.) not (animals not (humans and animals)).sh.

5. 3 and 4

4. Search strategy for CINAHL (EBSCOhost)

S1 ((MH "Respiratory Distress Syndrome, Acute") OR (MH "Respiratory Distress Syndrome") OR (MH "Acute Lung Injury")) OR (ARDS or ALI) OR ((Respiratory Distress Syndrome and (acute or adult)))

S2 (MM "Respiration, Artificial") OR TI (mechanical ventilation) OR TX lung protective ventilation strategy OR AB (protective and ventilation) OR TX LPVS

S3 S1 and S2

5. Search strategy for ISIWeb of Science

#1 TS=(adult respiratory distress syndrome or acute respiratory distress syndrome or acute lung injury or ards or ali)

#2 TS=(artificial respiration) or TS=(mechanical ventilation) or TS=(protective SAME ventilation) or TS= LPVS

#3 TS=(random* or placebo* or multicenter* or prospective) or TS=(trail* SAME (clinical or controlled))

#4 #1 and #2 and #3

Appendix 2. Summary of excluded articles

Year	Authors	Exclued Reasons
1979	John A.	Patients do not match
1983	Christian E.	No control group
1988	ALAN H	Data deficient
1988	Gilbert C	Data deficient
1989	Schapera A	Patients do not match
1990	JAMES M.	Patients do not match
1990	ALAN H	Data deficient
1992	Esen F	No relevant outcome
1993	Mercat A	No control group
1993	Putensen C	No control group
1993	Gattinoni L	No control group
1994	Martin R	No control group
1994	Pappert D	No control group
1994	Puybasset L	with Nitric oxide
1994	Sydow M	No control group
1994	Brunet F	No control group
1994	Jounieaux V	No relevant outcome
1995	Wickerts CJ	No control group
1995	Puybasset L	With Nitric oxide
1995	Samama CM	With Nitric oxide

1995	Ambrosino N	Study objective did not match ,Comparison of non-invasive ventilation
1995	Gentilello LM	Non-randomized trials
1996	Davis K Jr	Crossover study.
1996	Walmrath D	Study objective did not match
1996	Pelosi P	Study objective did not match
1996	Thorens JB	Non-randomized trials
1996	Wiswell TE	Patients do not match
1996	Hirschl RB	Case Report
1996	Fretschner R	Study objective did not match
1996	Kiiski R	Patients do not match
1996	Cereda M	Non-randomized trials
1996	Borelli M	Non-randomized trials
1996	Lundin S	with Nitric oxide
1996	Gerstmann DR	Patients do not match
1996	Messinger G	Non-randomized trials
1997	Tejeda M	Non-randomized trials
1997	C-M LIM,	Study objective did not match，
1997	Mourgeon E	With Nitric oxide
1997	Blanch L	With Nitric oxide
1997	Lewandowski K	Non-randomized trials
1997	Fort P	No control group
1997	Nava S	Non-randomized trials,Patients do not match
1997	Mergoni M	Non-randomized trials
1997	Troncy E	With Nitric oxide
1997	Warters RD	Non-randomized trials
1997	Mercat A	No control group
1997	Jellinek H	Non-randomized trials
1997	Artucio H	No control group
1997	Kalfon P	Non-randomized trials
1997	Blanch L	No control group
1997	Carvalho CR	part of eligible trial
1997	Iotti GA	No control group
1998	Alvarez A	No control group
1998	Genoni M	No control group
1998	Borelli M	No control group
1998	Jolliet P	Non-randomized trials
1998	Stewart TE	Patients do not match
1998	Gore DC	No control group
1998	Bersten AD	Non-randomized trials
1998	Croce MA	Non-randomized trials
1998	Hirschl RB	No control group

1998	Cohendy R	Case Report
1998	Cohendy R	Patients do not match
1998	Wood KA	Patients do not match
1999	Guerin C	Patients do not match,no control group
1999	Martinez M	No control group
1999	Baigorri F	No control group
1999	Voggenreiter G	No control group
1999	Cooper AB	Long-term follow-up
1999	Calderini E	Study objective did not match
1999	Xirouhaki N	No control group
1999	Lapinsky SE	No control group, Patients do not match
1999	Putensen C	Non-randomized trials
2000	Martin TJ	Patients do not match
2000	Wiedemann HP	Study objective did not match
2000	Confalonieri M	Communicate with the author
2000	Miro AM	Non-randomized trials
2000	Okamoto K	with Nitric oxide
2000	Okamoto K	No control group
2000	Nakagawa NK	Study objective did not match,Patients do not match
2000	Foti G	No control group
2000	Borelli M	Factorial analysis
2000	Kattwinkel J	Patients do not match
2000	Kondili E	No control group
2000	Masip J	Patients do not match
2000	Antonelli M	Patients do not match
2000	Tripathi M	Case Report
2000	Luhr OR	Patients do not match
2000	Hoffman LA	No control group
2000	Delclaux C	Patients do not match
2001	Staudinger T	with Nitric oxide
2001	Burns D	No control group
2001	Papazian L	Non-randomized trials, no control group
2001	Rocco M	No control group
2001	Schmitt JM	Non-randomized trials, no control group
2001	Johannigman JA	No control group, with Nitric oxide
2001	Mehta S	Non-randomized trials, no control group
2001	Crotti S	Non-randomized trials, no control group
2001	Davis K Jr	No control group
2001	Chiumello D	Patients do not match
2001	Hering R	No control group
2001	Conrad SA	No relevant outcome

2001	De Robertis E	No control group
2001	Wrigge H	Ungrouped，no relevant outcome
2001	Staudinger T	Patients do not match
2001	McKinley BA	Study objective did not match,
2001	Huang CC	Ungrouped, no control group
2001	Eisner MD	Secondary analysis of experimental ARDS Network
2001	van der Voort PH	Study objective did not match,
2001	Mercat A	No control group
2001	Huang CC	Non-randomized trials, no control group，Patients do not match
2002	Thys F	Comparison of non-invasive ventilation
2002	Confalonieri M,	Comparison of non-invasive ventilation
2002	Charles D.	Patients do not match
2002	Malhotra A	Communicate with the author
2002	Hering R	No control group
2002	Lee DL	Non-randomized trials
2002	Cam BV	Patients do not match
2002	Ward NS	Ungrouped
2002	Prin S	Non-randomized trials, no control group
2002	de Durante G	Non-randomized trials, no control group
2002	Matejovic M	Non-randomized trials, no control group
2002	Hering R	No relevant outcome
2002	Gregoretti C	No relevant outcome
2002	Da Conceiçao M	No control group
2002	Reper P	No relevant outcome
2002	Vieillard-Baron A	Non-randomized trials, no control group
2002	Richard JC	crossover study
2002	Blanc Q	Non-randomized trials, no control group
2002	Da Conceiçao M	No relevant outcome，no control group
2002	Pfeiffer B	Ungrouped
2002	Vieillard-Baron A	Non-randomized trials
2002	Tan PS	with Nitric oxide
2003	Mehta S	with Nitric oxide
2003	Kraincuk P	No control group
2003	Kuhlen R	No relevant outcome
2003	Park KJ	with Nitric oxide
2003	Varpula T	No relevant outcome
2003	Kwok H	Study objective did not match,
2003	Albaiceta GM	No relevant outcome
2003	Gerlach H	with Nitric oxide
2003	Lim CM	No relevant outcome
2003	Passam F	No control group

2003	Edibam C	No control group
2003	Brower RG	Non-randomized trials, no control group
2003	Tugrul S	No control group
2003	Prat G	No control group
2003	Boyer A	Study objective did not match,
2003	Bugedo G	No control group
2003	Dyhr T	No control group
2003	Fernández-Vivas M	Comparison of non-invasive ventilation
2003	Ferrer M	Comparison of non-invasive ventilation
2003	Cross AM	Patients do not match
2003	Gainnier M	No control group
2003	Easby J	No control group
2003	Schelling G	Study objective did not match,
2003	Richard JC	No control group
2003	Pelosi P	No control group
2003	Mehta S	No control group
2003	Schreiter D	Case Report
2004	Inal-Ince D	Study objective did not match,
2004	Fernández MD	No control group
2004	Bein T	Secondary analysis
2004	Martínez-Pérez M	No control group
2004	Guerin C	No homogenization
2004	Corrado A	Patients do not match
2004	Taylor RW	With Nitric oxide
2004	Cuomo A	Comparison of non-invasive ventilation
2004	Oczenski W	No relevant outcome
2004	Lam CF	No relevant outcome
2004	Ge Y	No relevant outcome
2005	Mascia L	No relevant outcome
2005	Zhang NX	No relevant outcome
2005	Ferguson ND	No control group
2005	Papazian L	No relevant outcome
2005	Gu Q	Non-randomized trials, no control group
2005	YI Li	No relevant outcome
2005	Albaiceta GM	No control group
2005	Kahn JM	No relevant outcome
2005	Patroniti N	No relevant outcome，no control group
2005	Honrubia T	Comparison of non-invasive ventilation
2005	L'Her E	Crossover study
2005	Sudarsanam TD	Study objective did not match,
2005	Varelmann D	Crossover study

2005	Oczenski W	Non-randomized trials, no control group
2005	Prebio M	Study objective did not match, no relevant outcome
2005	Tugrul S	Non-randomized trials, no control group
2005	Kallet RH	Crossover study
2006	Wurz J	Study objective did not match,
2006	Girgis K	Non-randomized trials, no control group
2006	Mancebo J	Comparison of non-invasive ventilation
2006	Schneider E	Crossover study
2006	Stahl CA	No control group
2006	M Reindl1,	No control group
2006	Rossetti HB	No control group
2006	Chiumello D	No control group
2006	Kallet RH	Non-randomized trials, no control group
2006	Brander L	Non-prospective study
2006	Roch A	No control group
2006	Lasocki S	No control group
2006	Galiatsou E	No control group
2006	Kallet RH	Non-randomized trials, no control group
2006	Kondili E	No control group
2006	Hinkson CR	No control group, Study objective did not match,
2006	Gregoretti C	Patients do not match
2007	Confalonieri M	Non-randomized trials,
2007	Bhandari V	Patients do not match
2007	Mentzelopoulos	Crossover study.
2007	Remick D	Communicate with the author
2007	Villar J	Ungrouped
2007	Coimbra VR	Patients do not match
2007	Grasso S	No control group
2007	Toublanc B	Cross-over study
2007	Talmor D	Cohort study
2007	Toth I	No control group
2007	McClintock DE	Secondary analysis of experimental ARDS Network
2007	Malhotra A	Case Report
2007	Galiatsou E	Communicate with the author
2007	Yang LY	Cross-over study
2008	Colombo D	No control group
2008	Arnal JM	Non-randomized trials, no control group
2008	Owens RL	No control group
2008	Muellenbach R.M.	Communicate with the author
2008	Kacmarek RM	Communicate with the author
2008	David M	Study objective did not match,

2008	Chiumello D	Study objective did not match,
2008	Wolthuis EK	Patients do not match
2008	Thalanany MM	Non-randomized trials, no control group
2008	Mireles-Cabodevila E	Mathematical model
2008	Demory D	No control group
2008	Constantin JM	Crossover study
2009	Koutsoukou A	No relevant outcome
2009	Mekontso Dessap A	No control group
2009	Darmon M	No relevant outcome
2009	WANG Yan	Comparison of non-invasive ventilation
2009	Michelet P	Comparison of non-invasive ventilation
2009	Agarwal R	Comparison of non-invasive ventilation
2009	Fraticelli AT	Study objective did not match,
2009	Gupta V	No relevant outcome
2009	Allardet-Servent J	No relevant outcome
2009	Bein T	Case Report
2009	Ling-ling Du	Study objective did not match,
2009	Lee D.L	Non-randomized trials, no control group
2009	Chung K	Patients do not match
2009	Carteaux G	No control group
2009	Cornejo R	No control group
2009	Malachias S	No relevant outcome
2009	Kokkoris S	No control group
2009	Mercat A	No relevant outcome
2009	Lowhagen K	No control group
2009	Piacentini E	Non-randomized trials, no control group
2009	Wang ZY	Study objective did not match,
2009	Sulemanji D	Lung model
2009	Badet M	No control group
2009	Cooke CR	Cost-effectiveness model
2009	Wu XY	Data deficient
2009	Tripathi M	Non-randomized trials, no control group
2009	Romero CM	Non-randomized trials, no control group
2009	Allardet-Servent J	No control group
2010	Maxwell RA	Patients do not match
2010	Constantin JM	Patients do not match
2010	Namendys-Silva SA	Communicate with the author
2010	Veelo DP	Study objective did not match,Ungrouped
2010	Iotti GA	Patients do not match
2010	Bajwa AA	No control group, crossover study
2010	Menadue C	Patients do not match， crossover study

2010	Crucean A.C	Patients do not match， no control group
2010	Mentzelopoulos	Crossover study，
2010	Montes FR	Study objective did not match,
2010	Isgrò S	No control group, crossover study
2010	Iannuzzi M	No relevant outcome
2010	Squadrone V	Study objective did not match,
2010	Kanoore Edul VS	No control group
2010	Iguchi N	Study objective did not match,
2010	Fougères E	No control group
2010	Briel M	Meta analysis
2010	Chung KK	Patients do not match
2010	Constantin JM	No control group
2010	Terzi N	No control group
2010	Schmidt M	No control group
2010	Verzilli D	Non-randomized trials， no relevant outcome
2010	Van de Louw A	No control group
2010	Di Marco F	Crossover study
2010	Papaioannou VE	Patients do not match
2010	Patroniti N	No control group
2010	Marraro GA	Study objective did not match,
2010	Boyer A	Crossover study, Patients do not match
2010	Pipeling MR	Case Report
2010	Patel DS	Patients do not match
2010	Carteaux G	Study objective did not match,
2010	Vaschetto R	Patients do not match
2010	Arnal J.-M	Crossover study
2010	Cebrian J	Cross-sectional study
2011	Mauri T	No control group
2011	MA Ling	Non-randomized trials, no control group
2011	Martin-Loeches I	Conference word
2011	El Fadl M.A	No control group
2011	Mauri T	Study objective did not match,
2011	Rodriguez P.O.	No control group
2011	Guervilly C	No control group
2011	Lang J	Patients do not match
2011	Sundaresan A	Non-randomized trials, no control group
2011	Mauri T	No control group
2011	Nemer SN	Study objective did not match,
2011	Sztrymf B	No control group
2011	Niwa T	Study objective did not match,
2011	Andrews PL	Crossover study， no control group

2011	Guo-hui Yang	No relevant outcome
2011	Chen CW	Non-randomized trials, no control group
2011	Ozsancak A	Study objective did not match,
2011	Zhang XY	No control group
2011	Cammarota G	No control group
2011	Funk GC	Patients do not match
2011	Bellani G	No control group
2011	Chiew YS	No control group
2011	Girault C	Patients do not match
2011	Nava S	Patients do not match
2011	Arnal JM	No control group
2011	Guinot PG	Study objective did not match,
2011	Dellamonica J	No control group
2011	Lowhagen K	Cross-over study
2011	Mentzelopoulos SD	No control group
2011	Leloup G	Case Report
2011	Liu W.-L	No relevant outcome
2012	Piquilloud L	No control group
2012	Mohamed M. Tawfeek	Comparison of non-invasive ventilation
2012	Carrillo A	Comparison of non-invasive ventilation
2012	Alkhuja S	Study objective did not match,
2012	ART Investigators	Protocol
2012	Franchi F	No relevant outcome
2012	Patroniti N	No control group
2012	Guervilly C	No control group
2012	Arnal JM	No control group
2012	Needham CJ	Study objective did not match,
2012	Forel JM	Study objective did not match,
2012	Vrettou C.S	Data deficient
2012	Mauri T	No control group
2012	Chiew Y.S	No control group
2012	Anjos CF	No control group
2012	Watkins TR	predictive model.
2012	Han SH	Not article or research
2012	Serpa Neto A	META analysis
2012	Sharaf M.S	No relevant outcome
2012	Guérin C	Non-randomized trials，
2012	Lucangelo U	Non-randomized trials, no control group
2012	Grasso S	Non-randomized trials
2012	Porot V	Non-randomized trials， no relevant outcome
2012	Bonastre J	Study objective did not match,

2012	Redondo Calvo FJ	Study objective did not match,
2012	de Matos GF	Non-randomized trials, no control group
2012	Küstermann J	Not article or research
2012	Smetkin AA	Study objective did not match,
2012	Katsiari M	No control group
2012	Gama de Abreu M,	Editorial
2013	Johnson RF Jr	Case Report
2013	Blum JM	Study objective did not match,
2013	Kowalski S	Study objective did not match,
2013	Schmidt M	No control group
2013	Natalini G	No control group
2013	Zhang JC	Study objective did not match,
2013	Dellamonica J	No control group
2013	Severgnini P	Patients do not match
2013	Huang Y	No control group
2013	Mauri T	No control group
2013	Rodriquez PO	No control group
2013	Schadler D	No relevant outcome
2013	Nava S	Patients do not match
2013	Mauri T	Non-randomized trials, no control group
2013	Rachmale S	Communicate with the author
2013	Retamal J	No relevant outcome
2013	Boissier F	Prevalence and prognosis of cor pulmonale
2013	Lhéritier G	Study objective did not match,
2013	Needham CJ	Study objective did not match,
2013	Vollman KM	No control group
2013	Wang WX	No relevant outcome
2013	Zhu GF	Patients do not match
2013	Wallet F	No control group
2013	Hoeper MM	No control group, Non-randomized trials
2013	Bertrand PM	Crossover study.
2013	Pintado M.C	Patients do not match
2013	James M.	Patients do not match
2013	Demet S.	Patients do not match
2014	Wang Xiuyan，	Patients do not match
2014	Yang Jing，	Non-randomized trials
2014	Jing Zhang	Retrospective study
2015	Emily Fish	Protocol
2015	Yaseen M.	Patients do not match

Appendix 3.

The all-cause mortality effect estimates from multiple treatment meta-analysis compared with direct and indirect estimates, based on back-calculated, and pair-wise meta-analyses.Direct and indirect estimates of effect and the corresponding Bayesian ‘I^2’ for inconsistency were calculated. And the ‘I^2’from Pooled pair-wise meta-analysis for heterogeneity were also calculated.

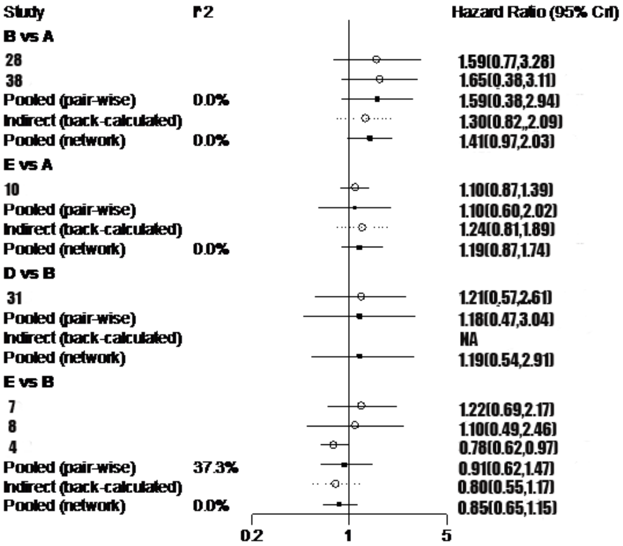

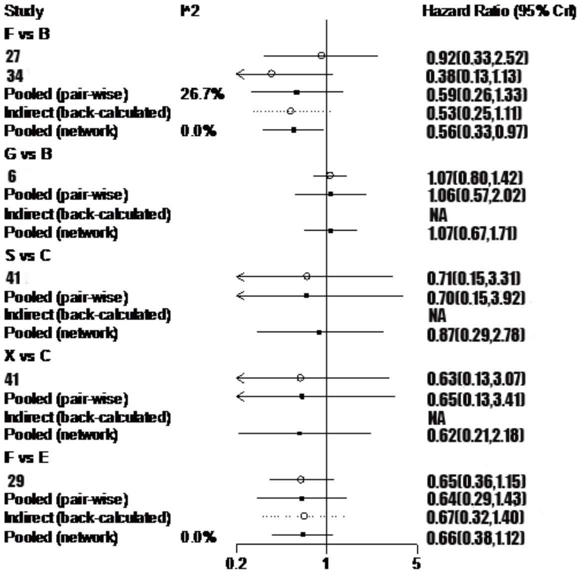

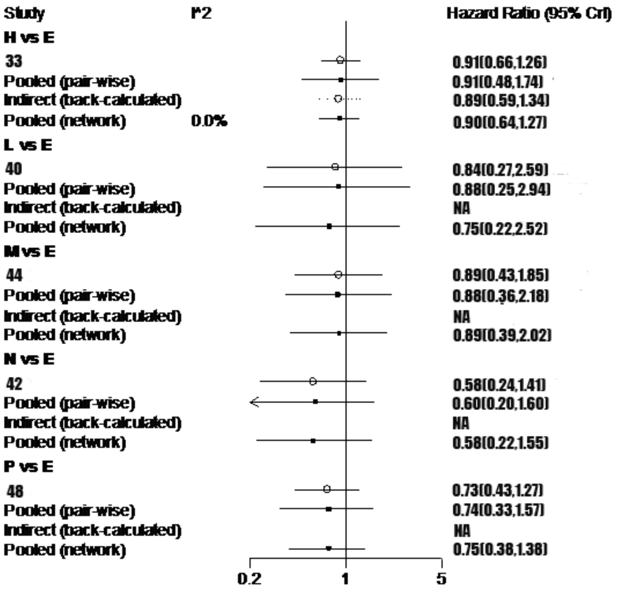
Study
I^2
Hazard Ratio (95% CrI)
H vs E
33
Pooled (pair-wise)
Indirect (back-calculated)
Pooled (network)
0.0%
0.91(0.66,1.26)
0.91(0.48,1.74)
0.89(0.59,1.34)
0.90(0.64,1.27)
L vs E
40
Pooled (pair-wise)
Indirect (back-calculated)
Pooled (network)
0.84(0.27,2.59)
0.88(0.25,2.94)
NA
0.75(0.22,2.52)
M vs E
44
Pooled (pair-wise)
Indirect (back-calculated)
Pooled (network)
0.89(0.43,1.85)
0.88(0.36,2.18)
NA
0.89(0.39,2.02)
N vs E
42
Pooled (pair-wise)
Indirect (back-calculated)
Pooled (network)
0.58(0.24,1.41)
0.60(0.20,1.60)
NA
0.58(0.22,1.55)
P vs E
48
Pooled (pair-wise)
Indirect (back-calculated)
Pooled (network)
0.73(0.43,1.27)
0.74(0.33,1.57)
NA
0.75(0.38,1.38)
0.2
1
5

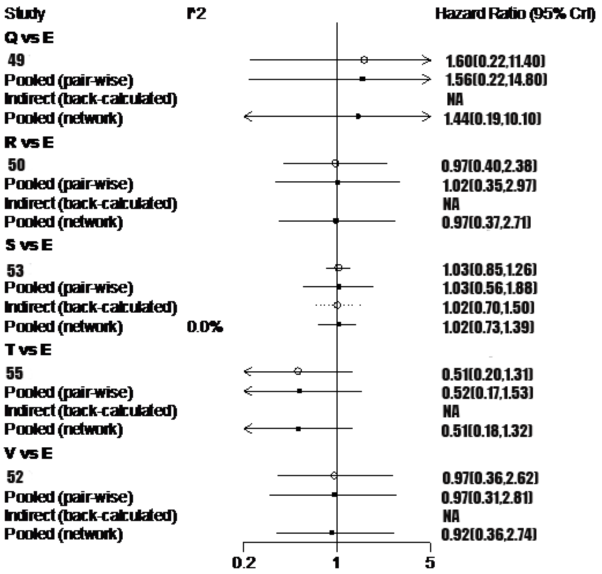
Study
I^2
Hazard Ratio (95% CrI)
Q vs E
49
Pooled (pair-wise)
Indirect (back-calculated)
Pooled (network)
1.60(0.22,11.40)
1.56(0.22,14.80)
NA
1.44(0.19,10.10)
R vs E
50
Pooled (pair-wise)
Indirect (back-calculated)
Pooled (network)
0.97(0.40,2.38)
1.02(0.35,2.97)
NA
0.97(0.37,2.71)
S vs E
53
Pooled (pair-wise)
Indirect (back-calculated)
Pooled (network)
0.0%
1.03(0.85,1.26)
1.03(0.56,1.88)
1.02(0.70,1.50)
1.02(0.73,1.39)
T vs E
55
Pooled (pair-wise)
Indirect (back-calculated)
Pooled (network)
0.51(0.20,1.31)
0.52(0.17,1.53)
NA
0.51(0.18,1.32)
V vs E
52
Pooled (pair-wise)
Indirect (back-calculated)
Pooled (network)
0.97(0.36,2.62)
0.97(0.31,2.81)
NA
0.92(0.36,2.74)
0.2
1
5

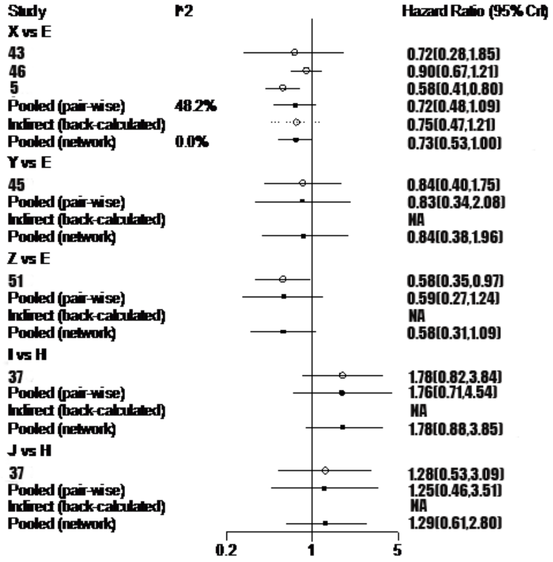
Study
I^2
Hazard Ratio (95% CrI)
X vs E
43
0.72(0.28,1.85)
46
0.90(0.67,1.21)
5
0.58(0.41,0.80)
Pooled (pair-wise) 48.2%
0.72(0.48,1.09)
Indirect (back-calculated)
0.75(0.47,1.21)
Pooled (network) 0.0%
0.73(0.53,1.00)
Y vs E
45
0.84(0.40,1.75)
Pooled (pair-wise)
0.83(0.34,2.08)
Indirect (back-calculated)
NA
Pooled (network)
0.84(0.38,1.96)
Z vs E
51
0.58(0.35,0.97)
Pooled (pair-wise)
0.59(0.27,1.24)
Indirect (back-calculated)
NA
Pooled (network)
0.58(0.31,1.09)
I vs H
37
1.78(0.82,3.84)
Pooled (pair-wise)
1.76(0.71,4.54)
Indirect (back-calculated)
NA
Pooled (network)
1.78(0.88,3.85)
J vs H
37
1.28(0.53,3.09)
Pooled (pair-wise)
1.25(0.46,3.51)
Indirect (back-calculated)
NA
Pooled (network)
1.29(0.61,2.80)
0.2
1
5

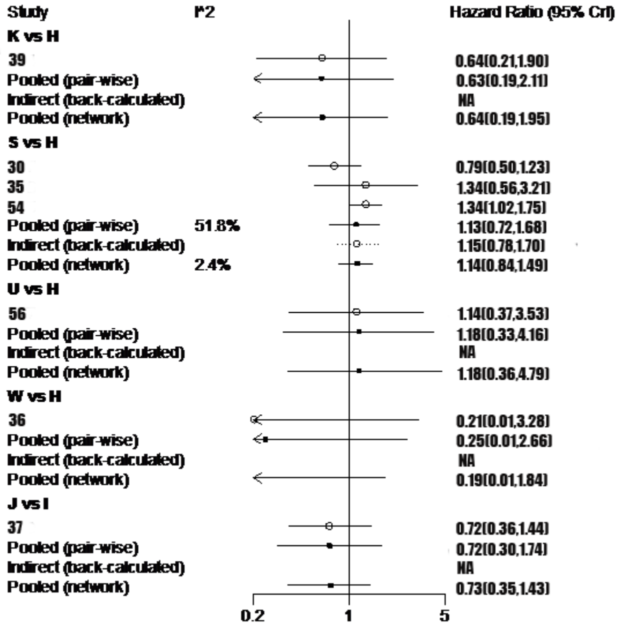
Study
I^2
Hazard Ratio (95% CrI)
K vs H
39
0.64(0.21,1.90)
Pooled (pair-wise)
0.63(0.19,2.11)
Indirect (back-calculated)
NA
Pooled (network)
0.64(0.19,1.95)
S vs H
30
0.79(0.50,1.23)
35
1.34(0.56,3.21)
54
1.34(1.02,1.75)
Pooled (pair-wise) 51.8%
1.13(0.72,1.68)
Indirect (back-calculated)
1.15(0.78,1.70)
Pooled (network) 2.4%
1.14(0.84,1.49)
U vs H
56
1.14(0.37,3.53)
Pooled (pair-wise)
1.18(0.33,4.16)
Indirect (back-calculated)
NA
Pooled (network)
1.18(0.36,4.79)
W vs H
36
0.21(0.01,3.28)
Pooled (pair-wise)
0.25(0.01,2.66)
Indirect (back-calculated)
NA
Pooled (network)
0.19(0.01,1.84)
J vs I
37
0.72(0.36,1.44)
Pooled (pair-wise)
0.72(0.30,1.74)
Indirect (back-calculated)
NA
Pooled (network)
0.73(0.35,1.43)
0.2
1
5

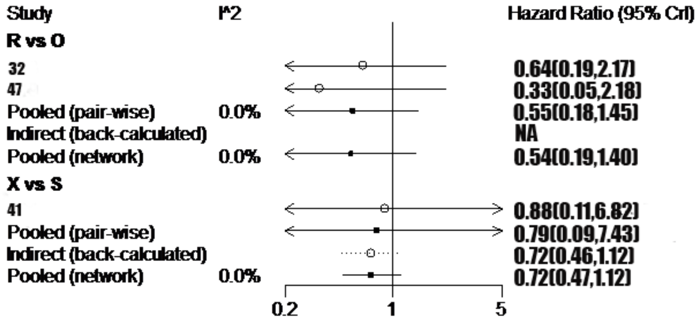
Study
I^2
Hazard Ratio (95% CrI)
R vs O
32
0.64(0.19,2.17)
47
0.33(0.05,2.18)
Pooled (pair-wise) 0.0%
0.55(0.18,1.45)
Indirect (back-calculated)
NA
Pooled (network) 0.0%
0.54(0.19,1.40)
X vs S
41
0.88(0.11,6.82)
Pooled (pair-wise)
0.79(0.09,7.43)
Indirect (back-calculated)
0.72(0.46,1.12)
Pooled (network) 0.0%
0.72(0.47,1.12)
0.2
1
5

Appendix 4. Rankings based on simulations in terms of all-cause mortality.

The numbers in the table represent the probability that each treatment is best (rank 1), the second highest (rank 2), etc. Rank probabilities sum to 1, both within a rank over treatments and within a treatment over ranks. The numbers in bold text represent the highest likelihood of ranking for each particular treatment.

	[,1]	[,2]	[,3]	[,4]	[,5]
A	2.17E-05	1.78E-04	5.75E-04	1.45 E-03	3.26 E-03
B	3.57E-04	3.84E-03	1.53E-02	4.15 E-02	8.18 E-02
C	7.75E-02	9.41E-02	8.68E-02	7.64 E-02	6.47 E-02
D	8.48E-02	1.14E-01	1.12E-01	1.01 E-01	8.60 E-02
E	5.00E-06	1.83E-05	2.05E-04	1.04 E-03	4.00 E-03
F	2.50E-05	1.62E-04	4.87E-04	1.15E-03	2.00 E-03
G	1.29E-02	3.88E-02	7.25E-02	9.97 E-02	1.15 E-01
H	3.33E-06	6.67E-05	4.22E-04	1.70E-03	4.71 E-03
I	1.04E-01	1.59E-01	1.58E-01	1.28E-01	9.79 E-02
J	1.43E-02	4.56E-02	7.34E-02	8.50 E-02	8.33 E-02
K	6.14E-03	1.28E-02	1.46E-02	1.74 E-02	1.82 E-02
L	2.84E-02	4.33E-02	4.31E-02	4.30 E-02	4.21 E-02
M	7.73E-03	1.87E-02	2.93E-02	3.65 E-02	4.16 E-02
N	1.58E-03	3.97E-03	6.67E-03	9.42 E-03	1.14 E-02
O	2.60E-01	1.65E-01	9.62E-02	6.97 E-02	5.34 E-02
P	8.42E-04	2.66E-03	5.61E-03	9.40 E-03	1.31 E-02
Q	2.92E-01	1.06E-01	6.65E-02	4.88 E-02	3.83 E-02
R	5.10E-03	4.16E-02	5.50E-02	5.73 E-02	5.71 E-02
S	1.35E-04	9.73E-04	3.61E-03	1.05E-02	2.28 E-02
T	1.15E-03	2.44E-03	4.14E-03	6.12 E-03	7.27 E-03
U	5.70E-02	7.34E-02	6.73E-02	6.06 E-02	5.37 E-02
V	2.93E-02	4.64E-02	5.32E-02	5.35 E-02	5.17 E-02
W	1.04E-02	1.08E-02	9.89E-03	9.17 E-03	8.73 E-03
X	3.33E-06	3.83E-05	9.33E-05	2.73 E-04	5.67 E-04
Y	5.93E-03	1.52E-02	2.38E-02	3.02 E-02	3.50 E-02
Z	1.13E-04	4.35E-04	9.82E-04	1.67 E-03	2.62E-03

	[,6]	[,7]	[,8]	[,9]	[,10]	[,11]
A	6.64 E-03	1.12 E-02	1.81 E-02	2.65 E-02	3.74 E-02	4.97E-02
B	1.22 E-01	1.47 E-01	1.50E-01	1.28 E-01	9.92 E-02	6.98 E-02
C	5.60 E-02	4.63 E-02	4.07 E-02	3.55 E-02	3.10 E-02	2.83E-02
D	7.09 E-02	5.90 E-02	4.83 E-02	3.99E-02	3.40E-02	2.94 E-02
E	1.12 E-02	2.82E-02	5.84E-02	1.00 E-01	1.39 E-01	1.67 E-01
F	3.42 E-03	5.28 E-03	7.19 E-03	9.97 E-03	1.28 E-02	1.66 E-02
G	1.13 E-01	1.04 E-01	8.79 E-02	7.01 E-02	5.53 E-02	4.44 E-02

H	1.01E-02	1.78 E-02	2.89 E-02	4.13 E-02	5.71E-02	7.35E-02
I	7.39 E-02	5.40E-02	4.18 E-02	3.33E-02	2.57 E-02	2.13 E-02
J	7.66 E-02	6.70E-02	5.82 E-02	5.10 E-02	4.54E-02	4.09 E-02
K	1.90 E-02	1.90 E-02	1.84 E-02	1.89 E-02	1.84 E-02	1.83 E-02
L	4.04 E-02	3.70 E-02	3.34 E-02	3.09 E-02	2.82 E-02	2.67E-02
M	4.49 E-02	4.49 E-02	4.29 E-02	4.18E-02	4.00E-02	3.98 E-02
N	1.34 E-02	1.49 E-02	1.54E-02	1.55 E-02	1.66 E-02	1.74 E-02
O	4.03 E-02	3.31 E-02	2.66 E-02	2.27E-02	1.97 E-02	1.73E-02
P	1.69 E-02	1.97 E-02	2.31 E-02	2.52 E-02	2.75 E-02	3.05 E-02
Q	3.12 E-02	2.69 E-02	2.35 E-02	2.03 E-02	1.71 E-02	1.54 E-02
R	5.29 E-02	4.96E-02	4.49E-02	4.19 E-02	3.74 E-02	3.55 E-02
S	4.11 E-02	6.46 E-02	8.84 E-02	1.07 E-01	1.21 E-01	1.18E-02
T	8.63 E-03	9.98E-03	1.08 E-02	1.13 E-02	1.22 E-02	1.28 E-02
U	4.87 E-02	4.18 E-02	3.71 E-02	3.35 E-02	2.98 E-02	2.81 E-02
V	4.86 E-02	4.51 E-02	3.97 E-02	3.72 E-02	3.40 E-02	3.20 E-02
W	8.55 E-03	7.91 E-03	7.32 E-03	7.29 E-03	6.20E-03	6.55 E-03
X	1.19 E-03	2.19 E-03	3.43E-03	5.56 E-03	8.66 E-03	1.33 E-02
Y	3.71 E-02	3.89 E-02	3.92 E-02	3.77 E-02	3.71E-02	3.74 E-02
Z	3.59E-03	4.80 E-03	5.87 E-03	7.21 E-03	8.80 E-03	1.03 E-02

	[,12]	[,13]	[,14]	[,15]	[,16]	[,17]
A	6.43E-02	8.00E-02	9.54E-02	1.07E-01	1.09E-01	1.04E-01
B	4.81E-02	3.41E-02	2.35E-02	1.54E-02	9.44E-03	5.33E-03
C	2.64E-02	2.55E-02	2.62E-02	2.68E-02	2.68E-02	2.67E-02
D	2.66E-02	2.51E-02	2.39E-02	2.26E-02	2.15E-02	1.91E-02
E	1.65E-01	1.37E-01	9.40E-02	5.42E-02	2.60E-02	1.05E-02
F	2.09E-02	2.70E-02	3.48E-02	4.42E-02	5.51E-02	6.81E-02
G	3.66E-02	3.04E-02	2.61E-02	2.23E-02	1.84E-02	1.45E-02
H	9.19E-02	1.08E-01	1.18E-01	1.18E-01	1.04E-01	8.21E-02
I	1.84E-02	1.59E-02	1.39E-02	1.20E-02	9.99E-03	8.06E-03
J	3.82E-02	3.71E-02	3.58E-02	3.53E-02	3.37E-02	3.15E-02
K	1.91E-02	2.03E-02	2.28E-02	2.56E-02	2.88E-02	3.21E-02
L	2.68E-02	2.80E-02	3.02E-02	3.24E-02	3.41E-02	3.70E-02
M	4.05E-02	4.21E-02	4.52E-02	4.64E-02	4.78E-02	4.87E-02
N	1.85E-02	2.05E-02	2.46E-02	2.76E-02	3.14E-02	3.60E-02
O	1.63E-02	1.59E-02	1.60E-02	1.61E-02	1.56E-02	1.48E-02
P	3.40E-02	3.87E-02	4.51E-02	5.20E-02	5.84E-02	6.48E-02
Q	1.49E-02	1.49E-02	1.55E-02	1.68E-02	1.64E-02	1.71E-02
R	3.41E-02	3.50E-02	3.60E-02	3.74E-02	3.94E-02	4.01E-02
S	1.09E-01	9.37E-02	7.41E-02	5.35E-02	3.70E-02	2.33E-02
T	1.42E-02	1.69E-02	1.92E-02	2.19E-02	2.58E-02	2.95E-02

U	2.71E-02	2.74E-02	2.86E-02	2.93E-02	3.02E-02	3.10E-02
V	3.15E-02	3.19E-02	3.37E-02	3.53E-02	3.59E-02	3.68E-02
W	6.32E-03	6.69E-03	7.17E-03	8.06E-03	8.96E-03	9.79E-03
X	2.05E-02	3.15E-02	4.67E-02	6.76E-02	9.29E-02	1.16E-01
Y	3.77E-02	3.89E-02	4.20E-02	4.52E-02	4.91E-02	5.05E-02
Z	1.32E-02	1.66E-02	2.15E-02	2.65E-02	3.37E-02	4.25E-02

	[,18]	[,19]	[,20]	[,21]	[,22]
A	8.98E-02	7.07E-02	5.30E-02	3.41E-02	2.04E-02
B	2.98E-03	1.45E-03	6.10E-04	2.63E-04	8.83E-05
C	2.67E-02	2.58E-02	2.61E-02	2.53E-02	2.62E-02
D	1.70E-02	1.53E-02	1.33E-02	1.18E-02	9.54E-03
E	2.91E-03	7.35E-04	1.42E-04	1.83E-05	6.67E-06
F	8.11E-02	9.37E-02	1.04E-01	1.11E-01	1.07E-01
G	1.15E-02	8.72E-03	6.20E-03	4.57E-03	3.17E-03
H	5.91E-02	3.86E-02	2.30E-02	1.21E-02	5.45E-03
I	6.40E-03	5.61E-03	4.03E-03	3.19E-03	2.55E-03
J	2.96E-02	2.66E-02	2.39E-02	2.05E-02	1.77E-02
K	3.54E-02	3.99E-02	4.59E-02	5.42E-02	6.61E-02
L	3.73E-02	4.01E-02	4.21E-02	4.58E-02	5.00E-02
M	5.01E-02	5.00E-02	4.97E-02	4.78E-02	4.54E-02
N	4.08E-02	4.76E-02	5.64E-02	6.73E-02	8.03E-02
O	1.42E-02	1.34E-02	1.33E-02	1.26E-02	1.25E-02
P	6.96E-02	7.61E-02	7.91E-02	7.87E-02	7.46E-02
Q	1.74E-02	1.84E-02	1.86E-02	2.07E-02	2.25E-02
R	4.02E-02	3.95E-02	4.18E-02	4.07E-02	3.94E-02
S	1.41E-02	7.96E-03	4.63E-03	2.33E-03	1.09E-03
T	3.52E-02	4.15E-02	5.01E-02	6.13E-02	7.89E-02
U	3.11E-02	3.20E-02	3.47E-02	3.48E-02	3.71E-02
V	3.76E-02	3.85E-02	3.93E-02	4.12E-02	4.10E-02
W	1.08E-02	1.24E-02	1.49E-02	1.78E-02	2.26E-02
X	1.34E-01	1.35E-01	1.19E-01	9.30E-02	5.98E-02
Y	5.24E-02	5.35E-02	5.32E-02	5.54E-02	5.47E-02
Z	5.33E-02	6.66E-02	8.37E-02	1.04E-01	1.22E-01

	[,23]	[,24]	[,25]	[,26]
A	1.09E-02	4.54E-03	1.55E-03	2.30E-04
B	3.33E-05	1.17E-05	1.67E-06	0.00E+00
C	2.47E-02	2.21E-02	1.98E-02	7.74E-03
D	6.67E-03	4.64E-03	2.60E-03	6.98E-04
E	0.00E+00	0.00E+00	0.00E+00	0.00E+00
F	9.20E-02	6.45E-02	3.12E-02	6.76E-03

G	2.06E-03	1.25E-03	6.03E-04	1.63E-04
H	2.10E-03	5.88E-04	8.00E-05	3.33E-06
I	1.53E-03	8.55E-04	3.90E-04	6.50E-05
J	1.36E-02	9.65E-03	4.96E-03	9.67E-04
K	8.47E-02	1.11E-01	1.55E-01	7.82E-02
L	5.58E-02	5.70E-02	5.88E-02	2.83E-02
M	3.93E-02	3.21E-02	2.08E-02	5.97E-03
N	9.82E-02	1.22E-01	1.41E-01	6.11E-02
O	1.20E-02	1.08E-02	9.07E-03	3.65E-03
P	6.63E-02	5.02E-02	3.03E-02	7.68E-03
Q	2.55E-02	3.09E-02	4.20E-02	2.21E-02
R	3.84E-02	3.19E-02	2.10E-02	6.76E-03
S	3.97E-04	1.32E-04	2.17E-05	0.00E+00
T	1.01E-01	1.37E-01	1.84E-01	9.65E-02
U	3.81E-02	4.01E-02	3.51E-02	1.26E-02
V	4.15E-02	4.18E-02	3.28E-02	1.05E-02
W	3.03E-02	4.61E-02	8.91E-02	6.16E-01
X	3.19E-02	1.33E-02	3.47E-03	3.72E-04
Y	5.13E-02	4.22E-02	2.85E-02	7.92E-03
Z	1.31E-01	1.25E-01	8.81E-02	2.54E-02

Appendix 5. Rankings based on simulations in terms of the incidence of barotraumas .

The numbers in the table represent the probability that each treatment is best (rank 1), the second highest (rank 2), etc. Rank probabilities sum to 1, both within a rank over treatments and within a treatment over ranks. The numbers in bold text represent the highest likelihood of ranking for each particular treatment.

	[,1]	[,2]	[,3]	[,4]	[,5]	[,6]
A	1.36E-03	4.49E-03	1.02E-02	1.92E-02	3.14E-02	4.60E-02
B	1.36E-02	5.19E-02	1.04E-01	1.57E-01	1.77E-01	1.64E-01
D	1.01E-01	1.23E-01	1.40E-01	1.26E-01	9.71E-02	7.74E-02
E	3.17E-04	2.42E-03	9.01E-03	2.61E-02	5.85E-02	1.07E-01
F	2.83E-03	7.93E-03	1.66E-02	2.71E-02	3.91E-02	5.04E-02
H	1.42E-03	8.11E-03	4.16E-02	8.98E-02	1.14E-01	1.18E-01
I	1.15E-01	2.63E-01	1.98E-01	1.07E-01	6.80E-02	4.81E-02
J	3.08E-01	2.92E-01	1.45E-01	7.25E-02	4.36E-02	3.03E-02
K	4.72E-04	7.12E-04	1.14E-03	1.27E-03	1.72E-03	1.51E-03
L	1.03E-01	8.84E-02	7.96E-02	6.62E-02	5.24E-02	4.60E-02
N	2.74E-01	1.19E-02	9.66E-03	6.18E-03	4.71E-03	3.94E-03

S	1.02E-02	2.86E-02	7.72E-02	1.06E-01	1.08E-01	9.86E-02
T	3.35E-02	5.45E-02	7.51E-02	8.33E-02	8.25E-02	7.84E-02
X	9.53E-03	2.22E-02	3.92E-02	5.48E-02	6.59E-02	7.36E-02
Y	2.58E-02	4.07E-02	5.36E-02	5.79E-02	5.69E-02	5.67E-02

	[,7]	[,8]	[,9]	[,10]	[,11]	[,12]
A	6.14E-02	8.01E-02	1.04E-01	1.36E-01	1.67E-01	1.73E-01
B	1.32E-01	9.22E-02	5.70E-02	3.16E-02	1.45E-02	4.81E-03
D	6.37E-02	5.33E-02	4.89E-02	4.72E-02	4.40E-02	3.83E-02
E	1.63E-01	2.02E-01	1.97E-01	1.41E-01	6.84E-02	2.18E-02
F	6.12E-02	7.31E-02	8.76E-02	1.11E-01	1.40E-01	1.68E-01
H	1.15E-01	1.07E-01	1.03E-01	1.01E-01	9.04E-02	6.86E-02
I	3.79E-02	3.24E-02	3.00E-02	2.78E-02	2.53E-02	2.23E-02
J	2.30E-02	1.99E-02	1.80E-02	1.55E-02	1.22E-02	9.80E-03
K	1.53E-03	1.69E-03	1.81E-03	2.32E-03	3.13E-03	4.89E-03
L	4.16E-02	4.09E-02	4.15E-02	4.69E-02	5.73E-02	7.59E-02
N	3.32E-03	3.23E-03	3.38E-03	3.86E-03	4.77E-03	6.33E-03
S	8.82E-02	8.15E-02	7.94E-02	8.08E-02	8.07E-02	7.61E-02
T	7.38E-02	7.10E-02	7.26E-02	7.79E-02	8.63E-02	9.16E-02
X	7.96E-02	8.44E-02	9.40E-02	1.05E-01	1.15E-01	1.18E-01
Y	5.61E-02	5.72E-02	6.17E-02	7.34E-02	9.01E-02	1.21E-01

	[,13]	[,14]	[,15]
A	1.27E-01	3.83E-02	1.06E-03
B	1.00E-03	9.67E-05	0.00E+00
D	2.98E-02	1.03E-02	4.25E-04
E	4.17E-03	3.48E-04	3.33E-06
F	1.59E-01	5.44E-02	1.84E-03
H	3.46E-02	7.56E-03	5.67E-05
I	1.93E-02	6.71E-03	1.77E-04
J	7.05E-03	2.32E-03	5.50E-05
K	1.10E-02	3.32E-01	6.34E-01

L	1.69E-01	8.64E-02	4.52E-03
N	1.23E-02	3.00E-01	3.52E-01
S	6.38E-02	2.11E-02	3.47E-04
T	8.66E-02	3.19E-02	1.04E-03
X	1.03E-01	3.52E-02	1.31E-03
Y	1.73E-01	7.30E-02	2.60E-03

Appendix 6.

Effect estimates for the incidence of barotraumas from multiple treatment meta-analysis compared with direct and indirect estimates, based on back-calculated, and pair-wise meta-analyses.

Direct and indirect estimates of effect and the corresponding Bayesian ‘I^2’ for inconsistency were calculated. And the ‘I^2’ from pooled pair-wise meta-analysis for heterogeneity were also calculated.

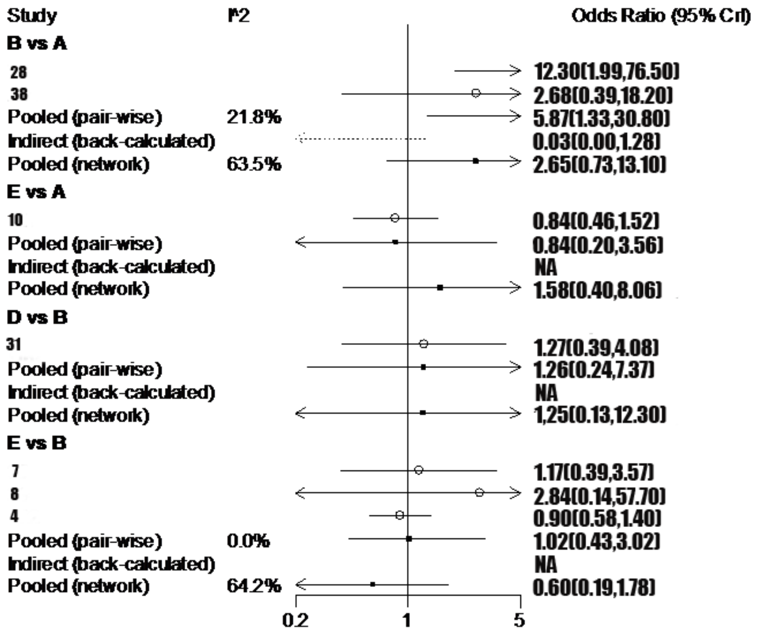

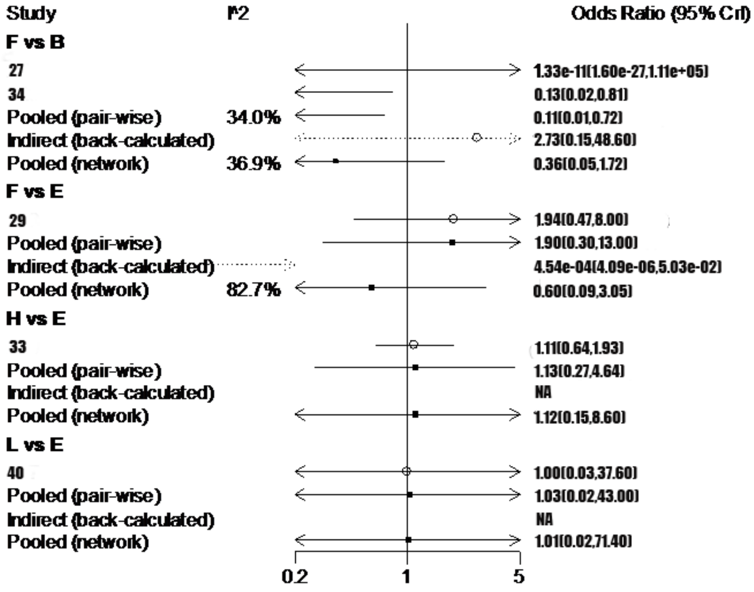
Study
I^2
Odds Ratio (95% CrI)
F vs B
27
1.33e-11(1.60e-27,1.11e+05)
34
0.13(0.02,0.81)
Pooled (pair-wise)
34.0%
0.11(0.01,0.72)
Indirect (back-calculated)
2.73(0.15,48.60)
Pooled (network)
36.9%
0.36(0.05,1.72)
F vs E
29
1.94(0.47,8.00)
Pooled (pair-wise)
1.90(0.30,13.00)
Indirect (back-calculated)
4.54e-04(4.09e-06,5.03e-02)
Pooled (network)
82.7%
0.60(0.09,3.05)
H vs E
33
1.11(0.64,1.93)
Pooled (pair-wise)
1.13(0.27,4.64)
Indirect (back-calculated)
NA
Pooled (network)
1.12(0.15,8.60)
L vs E
40
1.00(0.03,37.60)
Pooled (pair-wise)
1.03(0.02,43.00)
Indirect (back-calculated)
NA
Pooled (network)
1.01(0.02,71.40)
0.2
1
5

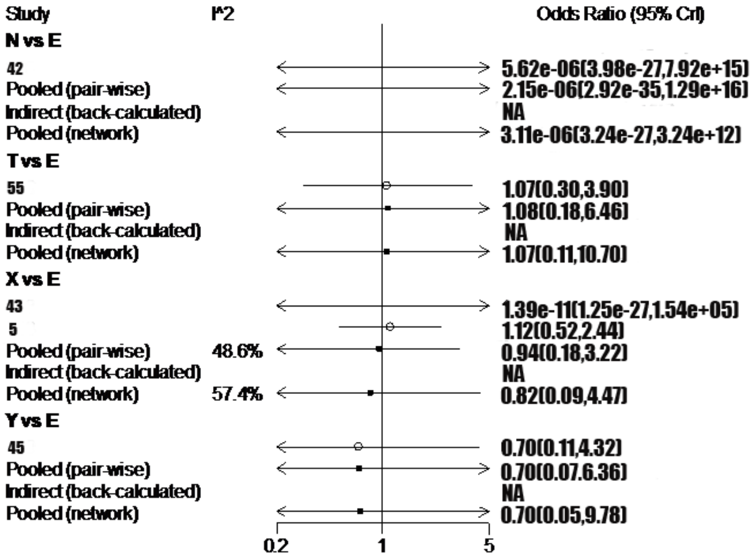
Study
I^2
Odds Ratio (95% CrI)
N vs E
42
5.62e-06(3.98e-27,7.92e+15)
Pooled (pair-wise)
2.15e-06(2.92e-35,1.29e+16)
Indirect (back-calculated)
NA
Pooled (network)
3.11e-06(3.24e-27,3.24e+12)
T vs E
55
1.07(0.30,3.90)
Pooled (pair-wise)
1.08(0.18,6.46)
Indirect (back-calculated)
NA
Pooled (network)
1.07(0.11,10.70)
X vs E
43
1.39e-11(1.25e-27,1.54e+05)
5
1.12(0.52,2.44)
Pooled (pair-wise)
48.6%
0.94(0.18,3.22)
Indirect (back-calculated)
NA
Pooled (network)
57.4%
0.82(0.09,4.47)
Y vs E
45
0.70(0.11,4.32)
Pooled (pair-wise)
0.70(0.07,6.36)
Indirect (back-calculated)
NA
Pooled (network)
0.70(0.05,9.78)
0.2
1
5

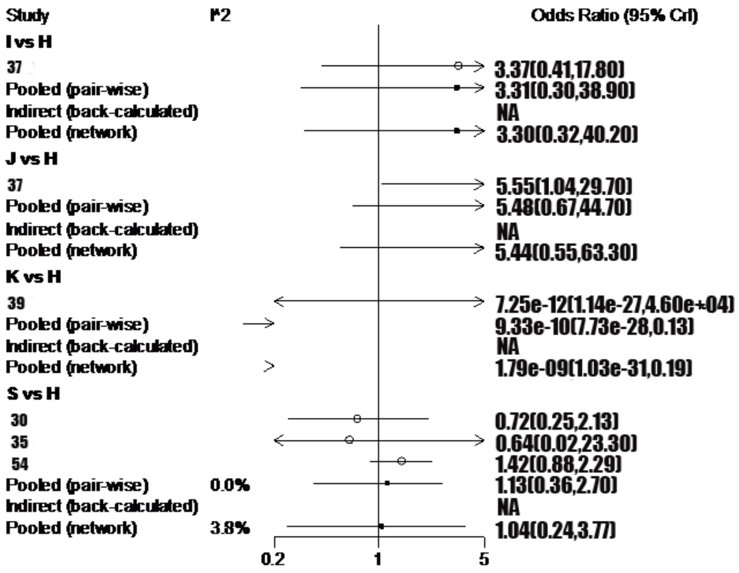
Study
I^2
Odds Ratio (95% CrI)
I vs H
37
3.37(0.41,17.80)
Pooled (pair-wise)
3.31(0.30,38.90)
Indirect (back-calculated)
NA
Pooled (network)
3.30(0.32,40.20)
J vs H
37
5.55(1.04,29.70)
Pooled (pair-wise)
5.48(0.67,44.70)
Indirect (back-calculated)
NA
Pooled (network)
5.44(0.55,63.30)
K vs H
39
7.25e-12(1.14e-27,4.60e+04)
Pooled (pair-wise)
9.33e-10(7.73e-28,0.13)
Indirect (back-calculated)
NA
Pooled (network)
1.79e-09(1.03e-31,0.19)
S vs H
30
0.72(0.25,2.13)
35
0.64(0.02,23.30)
54
1.42(0.88,2.29)
Pooled (pair-wise)
0.0%
1.13(0.36,2.70)
Indirect (back-calculated)
NA
Pooled (network)
3.8%
1.04(0.24,3.77)
0.2
1
5

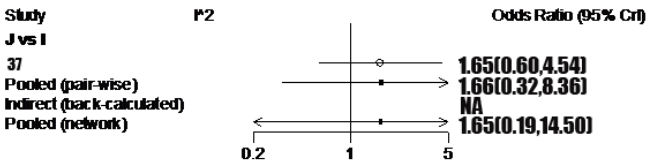
Study
I^2
Odds Ratio (95% CrI)
J vs I
37
1.65(0.60,4.54)
Pooled (pair-wise)
1.66(0.32,8.36)
Indirect (back-calculated)
NA
Pooled (network)
1.65(0.19,14.50)
0.2
1
5
